# Bio-convective and chemically reactive hybrid nanofluid flow upon a thin stirring needle with viscous dissipation

**DOI:** 10.1038/s41598-021-86968-8

**Published:** 2021-04-13

**Authors:** Arshad Khan, Anwar Saeed, Asifa Tassaddiq, Taza Gul, Poom Kumam, Ishtiaq Ali, Wiyada Kumam

**Affiliations:** 1 College of Aeronautical Engineering, National University of Sciences and Technology (NUST), Sector H-12, Islamabad, 44000 Pakistan; 2 Department of Mathematics, Abdul Wali Khan University, Mardan, Khyber, Pakhtunkhwa, 23200 Pakistan; 3 Department of Basic Sciences and Humanities, College of Computer and Information Sciences, Majmaah University, 11952, Al-Majmaah, Saudi Arabia; 4 Department of Mathematics, City University of Science and IT, Peshawar, 25000 KP Pakistan; 5 Center of Excellence in Theoretical and Computational Science (TaCS-CoE), Faculty of Science, King Mongkut’s University of Technology Thonburi (KMUTT), 126 Pracha Uthit Rd., Bang Mod, Thung Khru, Bangkok, 10140 Thailand; 6 Department of Medical Research, China Medical University Hospital, China Medical University, Taichung, 40402 Taiwan; 7 Department of Mathematics and Statistics, College of Science, King Faisal University, P. O. Box 400, Hafouf, Al- Ahsa, 31982 Saudi Arabia; 8 Program in Applied Statistics, Department of Mathematics and Computer Science, Faculty of Science and Technology, Rajamangala University of Technology Thanyaburi, Thanyaburi, Pathumthani, 12110 Thailand

**Keywords:** Applied mathematics, Nanoparticles

## Abstract

In this work, the thermal analysis for bio-convective hybrid nanofluid flowing upon a thin horizontally moving needle is carried out. The chemical reaction and viscous dissipation has also considered for flow system in the presence of microorganism. The hybrid nanoparticles comprising of Copper $\left( {Cu} \right)$ and Alumina $\left( {Al_{2} O_{3} } \right)$ are considered for current flow problem. Mathematically the flow problem is formulated by employing the famous Buongiorno’s model that will also investigate the consequences of thermophoretic forces and Brownian motion upon flow system. Group of similar variables is used to transform the model equations into dimensionless form and have then solved analytically by homotopy analysis method (HAM). It has established in this work that, flow of fluid declines due to increase in bioconvection Rayleigh number, buoyancy ratio and volume fractions of nanoparticles. Thermal flow grows due to rise in Eckert number, Brownian, thermophoresis parameters and volume fraction of nanoparticles. Concentration profiles increase due to growth in Brownian motion parameter and reduces due to increase in thermophoresis parameter and Lewis number. Motile microorganism profile declines due to augmentation in Peclet and bioconvection Lewis numbers. Moreover, the percentage enhancement in the drag force and rate of heat transfer using conventional nanofluid and hybrid nanofluid are observed and discussed. The hybrid nanofluid increases the skin friction and heat transfer rate more rapidly and efficiently as compared to other traditional fluids. A comparison of the present study with the existing literature is also conducted with a closed agreement between both results for variations in thickness of the needle.

## Introduction

The suspension of small sized particles (with size less than 100 nm) in a base/pure fluid for instance oil, water and ethylene glycol etc. is named as nanofluid while the small sized particles are named as nanoparticles such as silver $\left( {Ag} \right)$ , alumina $\left( {Al_{2} O_{3} } \right)$, copper $\left( {Cu} \right)$ and copper oxide $\left( {CuO} \right)$ etc. Since these fluids augment the thermal conductivity and improve the capability of heat transmission of pure fluid, so these fluids play a vital role as coolant in heat transmission equipment. These fluids also play a significant role in industry and engineering applications such as microelectronics, biomedical devices, vehicle cooling and power generation etc. Choi^1^ was the first gentleman who has suggested the number of nanoparticles in a pure fluid for augmenting the heat transfer characteristics of base fluid. After this work a number of researchers have diverted their attention to discuss the heat transmission characteristics of nanofluids by using different geometries and under different flow conditions. Khan et al.^2^ have discussed minimization of entropy production for a nanofluid past a thin needle by using thermal radiation. In this work the entropy production has analyzed through second law of thermodynamics. Salleh et al.^3^ have investigated numerically the stability analysis for hydromagnetic liquid motion upon a thin needle. In this work the model of Buongiorno has employed to investigate the impact of Brownian and thermoporetic forces upon flow system. Waini et al.^4^ have discussed the prescribed heat flux for a hybrid nanofluid over a vertical needle. In this work the modeled equations have been transformed to dimensionless form by using set of transformable variables and then have solved the resultant equations numerically by employing MATLAB software. Gul et al.^5^ have discussed nanofluid flow over thin needle for fractional order convective nanotubes.

During the past few decades, the deliberation on the theme of thin heated needle has achieved a considerable attention from a number of scientists and researchers due to its importance and contribution at industrial level. The work of Lee^6^ as comprehended by Narain and Uberoi^7^ measured the forced as well as free convective heat transmission past a vertical thin needle for a viscous liquid. In this extended work, the authors have investigated locally similar and series solutions for considered flow problem. Chen and Smith^8^ have discussed the mixed convective transfer of heat upon a thin needle. In this work the thermal characteristics are examined under the impacts of Prandtl number and size of needle for accelerating and uniform flow of liquid. Wang^9^ investigated numerically mixed convective flow of fluid upon a heated tip of vertically placed needle. Afterwards various investigations have been conducted for fluid flow upon a thin needle using various flow conditions^10–15^. Ramesh et al.^16^ have discussed thermal examination for hybrid nanofluid flow upon a thin needle using Darcy-Firchheimer porous surface characteristics and external heat source. The authors of this article have transformed the modeled equations into dimensionless form and then have determined a numerical solution for that set of dimensionless equations. Hashim et al.^17^ have discussed the thermophoresis properties for nanofluid flowing upon a continuously moving needle. In this article the investigation has carried out by employing viscous dissipation and the solution of modeled equations has carried out numerically by using Matlab software.

Transportation behavior in nanofluid is described by two models one model is suggested by Buongiorno^18^ while the other model is proposed by Tiwari and Das^19^. The model of Buongiorno is two components non-homogeneous in which slip velocity of pure/base fluid and nanoparticles is non-zero. This model has determined seven different slip mechanisms for nanofluid flow. These mechanisms are described as gravity, Magnus effect, Brownian motion, inertia, drainage of fluid, diffusiophoresis and thermophoretic effects. It has also proposed in Boungiorno’s work^18^ that out of the seven slip mechanisms only thermoporesis and Brownian diffusions are important for fluids containing nanoparticles. Due to importance of Buongiorno’s model numerous researchers have used this model successfully by using different geometries and various flow conditions. After a few years, Nield and Kuznetsov^20,21^ have expanded the Buongiorno’s work by taking some new conditions applied on boundary of the problem. These two authors have utilized the thermophoretic and Brownian motion terms into concentration and energy equations in order to investigate the impact of Brownian motion and thermophresis upon these equations. Afterwards, a number of researchers have used Buongiorno’s model for fluid flow by using different geometries and various flow conditions. Khan et al.^22,23^ have discussed the Buongiorno’s model for nanofluid flow upon a horizontal cylinder by using different flow conditions. The authors of these investigations have transformed the modeled equations into dimensionless form by employing the suitable sets of similar variables and then have solved the resultant equations by using semi-analytical technique HAM. The readers can further study about Buongiorno’s model for fluid flow by using different geometries and various flow conditions in Refs.^24–28^.

The occurrence of bio-convection is another striking field which consists of a number of physical and real world applications. The convective movement of a material due to gradient of density at microscopic level is termed as bio-convection. This instability in density gradient occurred due to collective swimming of microorganisms. Normally this phenomenon takes place at the upper most level of liquid due to which the liquid in that specific region become denser. Instability in flow system also occurs due to the segregation in density of the upper and lower level of liquid. There are numerous medical and biological processes that necessitate this physical phenomenon, for instance, bio-fuels, enzymes, micro-system, biological tissues, bacteria and bio-technology etc. The bioconvection process is categorized into different categories such as gyrotactic microorganism, chemotaxis and geotactic microorganisms. This categorization is based upon the directional movements of various microorganisms. Kuznetsov^29,30^ has investigated the bioconvection by using various types of nanoparticles. Mallikarjuna Mallikarjuna et al.^31^ discussed the steady biocovective flow for a nanofluid with gyrotactic microorganism over a vertical cylinder and have transformed the modeled problem into dimensionless form by using dimensionless variables and then have solved resultant equations in numerical form by finite difference method. Uddin et al.^32^ have discussed numerically the mathematical model to check the impacts of velocity slip of second order past a horizontal permeable plate. Chebyshev method has used in this investigation for approximate solution of problem. The reader can further study about the bioconvection fluid flow with different geometries and flow conditions in Refs.^33–37^.

Most of the modeled problems in the universe are extremely nonlinear and are also highly complex in nature to determine their solution; even sometimes the determination of exact solution of such problems is impossible. For the purpose of determination the solution of such complex problems, there is a need to use a specific analytical, semi analytical or numerical technique. HAM is one of such famous semi-analytical technique that is used to determine solution to highly nonlinear problems. This method was first introduced by Liao^38,39^ for solution of numerous nonlinear problems. This technique provides solution in functional form and is also very fast convergent method. The solution provided by this method involves all the parameters encountered in the physical modeling of the problem; hence the impact of these substantial parameters upon flow system can be discussed easily.

From above cited literature, we observe that many investigations have conducted for fluid flow upon a thin needle by using various flow conditions, but only one study^40^ has been found in literature that discussed the fluid flow with motile microorganism upon a thin moving needle. In this work^40^ traditional nanofluid has used. In our work we have considered the thermal analysis for bioconvection of hybrid nanofluid flowing upon a thin horizontally moving needle. The novelty of this work is increased further by considering the chemical reaction and viscous dissipation with gyrotactic microorganism for hybrid nanofluid which has not been discussed yet. Moreover, the hybrid nanoparticles comprising of Copper and Alumina are considered for current flow problem which further increase the originality of this work. For determination the impact of thermophoretic forces and Brownian motion upon flow system the famous Buongiorno’s model has also used in this study. After converting the modeled equations into dimensionless form, the popular semi-analytical technique (HAM) has used to determine the solution of resultant equations.

## Problem formulation

**Figure 1:**
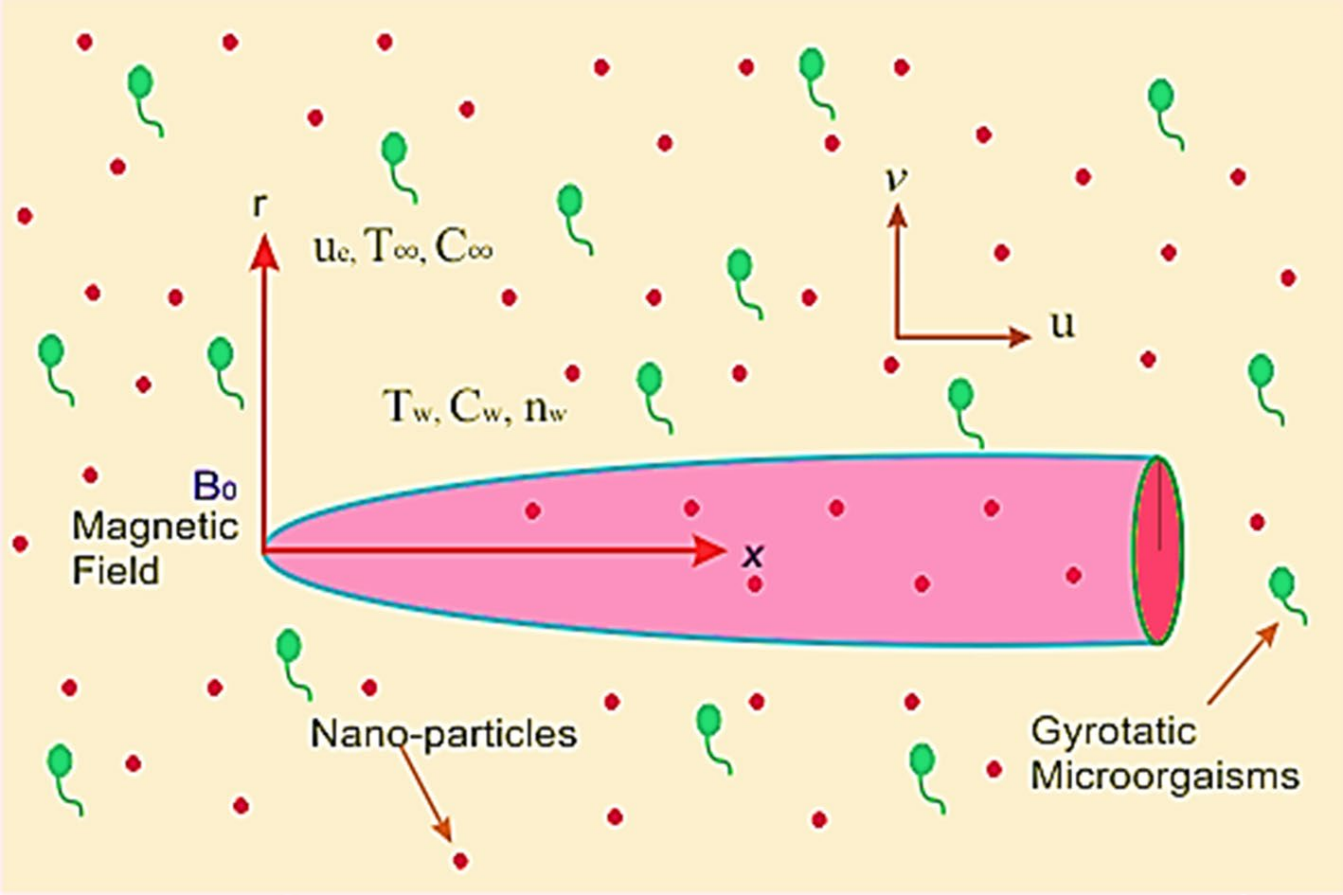
Geometry of flow problem.

Take a thin horizontal needle enclosed by a laminar viscous incompressible hybrid nanofluid. The hybrid nanoparticles comprise of Copper $\left( {Cu} \right)$ and Alumina $\left( {Al_{2} O_{3} } \right)$ while the base fluid is considered as water. Let $u,\,v$ be the flow components in $x$ as axial and $r$ as radial directions respectively as depicted in Fig. 1. Following assumptions are used to model the problem:

1. The flow is forced convective.
2. The needle is moving horizontally with uniform velocity $u_{w}$ in opposite or similar direction of surrounded fluid flowing upon the needle with fixed velocity $u_{\infty }$.
3. The radius of needle is $R\left( x \right) = \left( {\upsilon_{f} cx/U} \right)^{1/2}$ with $c$ as its size and $\upsilon_{f}$ as kinematic viscosity. Moreover, $U = u_{w} + u_{\infty }$ is composite velocity for current flow system.
4. The temperature, concentration and microorganism at needle surface are $T_{w} ,\,C_{w}$ and $n_{w}$ respectively while $T_{\infty } ,\,\,C_{\infty }$ and $n_{\infty }$ are their corresponding values for ambient fluid with $T_{w} > T_{\infty }$, $C_{w} > C_{\infty }$ and $n_{w} > n_{\infty }$.
5. The model of Buongiorno is employed to flow system with chemically reactive and viscous dissipative effects.

Considering all the above assumptions we have^40–42^:

1 $$ \frac{{\partial \left( {rv} \right)}}{\partial r} + \frac{{\partial \left( {ru} \right)}}{\partial x} = 0 $$

2 $$ u\,\,\frac{\partial u}{{\partial x}} + v\,\,\frac{\partial u}{{\partial r}} = \frac{{\mu_{hnf} }}{{\rho_{hnf} }}\,\,\frac{1}{r}\,\,\frac{\partial }{\partial r}\left( {r\,\,\frac{\partial u}{{\partial r}}} \right) + \frac{1}{{\rho_{hnf} }}\left\{ {\left( {1 - C_{\infty } } \right)\,\,\beta g\left( {T - T_{\infty } } \right) - \left( {\rho_{p} - \rho_{f} } \right)\left( {C - C_{\infty } } \right) - g\gamma \left( {\rho_{m} - \rho_{f} } \right)\left( {n - n_{\infty } } \right)} \right\} $$

3 $$ \left( {\rho C_{p} } \right)_{hnf} \left( {u\frac{\partial T}{{\partial x}} + v\frac{\partial T}{{\partial r}}} \right) = \kappa_{hnf} \frac{1}{r}\frac{\partial }{\partial r}\left( {r\frac{\partial T}{{\partial r}}} \right) + \tau \left( {D_{B} \,\,\frac{\partial C}{{\partial r}}\,\,\frac{\partial T}{{\partial r}}\,\, + \,\,\frac{{D_{T} }}{{T_{\infty } }}\,\,\left( {\frac{\partial T}{{\partial r}}} \right)^{2} } \right) + \mu_{hnf} \left( {\frac{\partial u}{{\partial r}}} \right)^{2} $$

4 $$ u\,\,\frac{\partial C}{{\partial x}} + \,\,v\,\,\frac{\partial C}{{\partial r}}\,\, = \,\,\frac{{D_{B} }}{r}\,\,\frac{\partial }{\partial r}\,\,\left( {r\,\,\frac{\partial C}{{\partial r}}} \right)\,\, + \,\,\frac{{D_{T} }}{{T_{\infty } }}\,\,\frac{1}{r}\,\,\frac{\partial }{\partial r}\,\,\left( {r\,\,\frac{\partial T}{{\partial r}}} \right) - K^{*} \left( {C - C_{\infty } } \right) $$

5 $$ u\,\,\frac{\partial n}{{\partial x}}\,\, + \,\,v\,\,\frac{\partial n}{{\partial r}} + \,\,\frac{{b\,\,W_{c} }}{{\left( {C_{w} - C_{\infty } } \right)}}\left[ {\frac{\partial }{\partial r}\,\,\left( {n\,\,\frac{\partial C}{{\partial r}}} \right)} \right] = D_{n} \,\,\frac{1}{r}\,\,\frac{\partial }{\partial r}\,\,\left( {r\frac{\partial n}{{\partial r}}} \right) $$

Above in Eqs. (1) – (5) $u,\,v$ depict the axial and radial components of flow. $T$ is temperature $C$ is concentration, $\rho_{hnf} ,\mu_{hnf} ,\kappa_{hnf} \,$ are respective representations for density, viscosity and thermal conductivity of hybrid nanofluid. The Brownian and themophoretic diffusion constant are denoted by $D_{B} ,\,D_{T}$ while $K^{*} = K_{0} /x$ is the rate of dimensionless reaction.

The required boundary conditions are^12,41^

6 $$ \begin{aligned} & u\left( {x.\,r} \right) = U_{w} ,\,\,v\left( {x.\,r} \right) = 0,\,\,T\left( {x.\,r} \right) = T_{w} ,\,\,C\left( {x.\,r} \right) = C_{w} ,\,\,n\left( {x,r} \right) = n_{w} \,\,\,\,\,at\,\,\,\,\,r = R\left( x \right), \\ & u\left( {x.\,r} \right) \to U_{\infty } ,\,\,\,\,T\left( {x.\,r} \right) \to T_{\infty } ,\,\,\,\,\,\,C\left( {x.\,r} \right) \to C_{\infty } ,\,\,\,\,\,n\left( {x,r} \right) \to n_{\infty } \,\,\,\,\,\,\,\,\,\,\,\,\,as\,\,\,\,\,r \to \infty \\ \end{aligned} $$

For the purpose of non-dimensionalization following group of similarity transformation has defined^12^

7 $$ \psi = v_{f} x.f\left( \eta \right),\,\,\,\,\eta = \frac{{Ur^{2} }}{{v_{f} x}},\,\,\,\,\theta \left( \eta \right) = \frac{{T - T_{\infty } }}{{T_{w} - T_{\infty } }},\,\,\,\phi \left( \eta \right) = \frac{{C - C_{\infty } }}{{C_{w} - C_{\infty } }},\,\,\xi \left( \eta \right) = \frac{{n - n_{\infty } }}{{n_{w} - n_{\infty } }} $$

The flow system for current work is streamlined, so in Eq. (7) $\psi$ is a stream function. The corresponding flow components for the assumed stream function are defined as below^12^

8 $$ u = \frac{1}{r}\frac{\partial \psi }{{\partial r}} = 2uf^{\prime } \left( \eta \right),\,\,\,\,\,\,\,v = - \frac{1}{r}\frac{\partial \psi }{{\partial x}} = - \frac{{\upsilon_{f} }}{r}\left( {f\left( \eta \right) - \eta f^{\prime } \left( \eta \right)} \right) $$

Next we shall incorporate Eq. (7) into Eqs. (1) – (5) so that after simplification we shall have

9 $$ \frac{2}{{\left( {1 - \varphi_{1} } \right)^{2.5} \left( {1 - \varphi_{2} } \right)^{2.5} }}\left( {\eta f^{\prime \prime \prime } + f^{\prime \prime } } \right) + \left\{ {\left( {1 - \varphi_{2} } \right)\left( {\left( {1 - \varphi_{1} } \right) + \varphi_{1} \frac{{\rho_{s1} }}{{\rho_{f} }}} \right) + \varphi_{2} \frac{{\rho_{s2} }}{{\rho_{f} }}} \right\}ff^{\prime \prime } + \lambda \left( {\theta - Nr\phi - R_{b} \xi } \right) = 0 $$

10 $$ \begin{aligned} & 2\frac{{k_{hnf} }}{{k_{f} }}\left( {\theta^{\prime } + \eta \theta^{\prime \prime } } \right) + \left\{ {\left( {1 - \varphi_{2} } \right)\left( {\left( {1 - \varphi_{1} } \right) + \varphi_{1} \frac{{\left( {\rho C_{p} } \right)_{s1} }}{{\left( {\rho C_{p} } \right)_{f} }}} \right) + \varphi_{2} \frac{{\left( {\rho C_{p} } \right)_{s2} }}{{\left( {\rho C_{p} } \right)_{f} }}} \right\}\Pr f\theta^{\prime } \\ & \quad \quad \quad + \Pr Ec\frac{\eta }{{\left( {1 - \varphi_{1} } \right)^{2.5} \left( {1 - \varphi_{2} } \right)^{2.5} }}\left( {f^{\prime \prime } } \right)^{2} + 2\eta \Pr \left( {N_{b} \theta^{\prime } \phi^{\prime } + N_{t} \left( {\theta^{\prime } } \right)^{2} } \right) = 0 \\ \end{aligned} $$

11 $$ 2\left( {\phi^{\prime } + \eta \phi^{\prime \prime } } \right) + 2\frac{{N_{t} }}{{N_{b} }}\left( {\theta^{\prime } + \eta \theta^{\prime \prime } } \right) + Le\phi^{\prime } - \frac{1}{2}LeK\phi = 0 $$

12 $$ 2\left( {\xi^{\prime } + \eta \xi^{\prime \prime } } \right) + \Pr L_{b} \xi f^{\prime } - 2P_{e} \left( {2\eta \xi \phi^{\prime \prime } + \left( {\xi + \eta \xi^{\prime } } \right)\phi^{\prime } } \right) = 0 $$

Notice that the in above equations the prime notations depict the derivative with respect to similarity variable $\eta$. By considering $\eta = c$ that represents the needle wall, while the surface of needle is expressed as^12^

13 $$ R\left( x \right) = \left( {\frac{{\upsilon_{f} cx}}{U}} \right)^{1/2} $$

In this work the nanoparticles of copper and alumina are suspended in water which is taken as pure fluid. In order to obtain the hybrid nanofluid $Cu - Al_{2} O_{3} /H_{2} O$, first the nanoparticles of $Al_{2} O_{3}$ with volumetric fraction $\varphi_{1}$ are suspended in water this produced $Al_{2} O_{3} /H_{2} O$. Afterwards, nanoparticles of $Cu$ with volumetric fraction $\varphi_{2}$ are mixed in $Al_{2} O_{3} /H_{2} O$. This physical phenomenon will finally give us hybrid nanofluid $Cu - Al_{2} O_{3} /H_{2} O$. Moreover, in Eqns. (9–12) $\kappa_{s1} ,\,\,\rho_{s1} ,\,\,\left( {\rho C_{p} } \right)_{s1} ,\varphi_{1}$ are representations for thermal conductivity, density, heat capacity and volumetric fraction for $Al_{2} O_{3} - nanoparticles$ while $\kappa_{s2} ,\,\,\rho_{s2} ,\,\,\left( {\rho C_{p} } \right)_{s2} ,\varphi_{2}$ are the corresponding values for $Cu - nanoparticles$.

The dimensionless form of subjected BCs is

14 $$ \begin{aligned} & f\left( c \right) = \frac{\varepsilon }{2}c,\,\,\,\,f^{\prime } \left( c \right) = \frac{\varepsilon }{2},\,\,\,\theta \left( c \right) = 1,\,\,\,\,\phi \left( c \right) = 1,\,\,\xi \left( c \right) = 1, \\ & f^{\prime } \left( \infty \right) \to \frac{1}{2}\left( {1 - \varepsilon } \right),\,\,\,\theta \left( \infty \right) \to 0,\,\,\,\phi \left( \infty \right) \to 0,\,\,\xi \left( \infty \right) \to 0 \\ \end{aligned} $$

In above equations the dimensionless parameters $Nr$ is buoyancy ratio parameter, $R_{b}$ is bioconvection Rayleigh number, $\lambda$ is mixed convection parameter, $\Pr$ is Prandtl number, $N_{b}$ is Brownian motion parameter, $N_{t}$ is thermophoresis parameter, Eckert number is given by $Ec$, $Le$ is Lewis number, $K$ is chemical reaction parameter, $\varepsilon$ is velocity ratio parameter, $P_{e}$ is Peclet number and $L_{b}$ bioconvection Lewis number. Mathematically these parameters are defined as follows^12,40–42^:

15 $$ \begin{aligned} & N_{r} = \frac{{\left( {\rho_{p} - \rho_{f} } \right)\left( {C_{w} - C_{\infty } } \right)}}{{\rho_{f} \beta \left( {1 - C_{\infty } } \right)\left( {T_{w} - T_{\infty } } \right)}},\,\,\,\,\,\,R_{b} = \frac{{\left( {\rho_{m} - \rho_{f} } \right)\left( {n_{w} - n_{\infty } } \right)}}{{\rho_{f} \beta \left( {1 - C_{\infty } } \right)\left( {T_{w} - T_{\infty } } \right)}},\,\,\, \\ & \lambda = \frac{{Gr_{x} }}{{{\text{Re}}_{x}^{2} }}, \,\Pr = \frac{{\upsilon_{f} \left( {\rho C_{p} } \right)_{hnf} }}{{k_{f} }},\,\,\,\, \\ & N_{b} = \frac{{\tau D_{B} \left( {C_{w} - C_{\infty } } \right)}}{{\upsilon_{f} }},\,\,\,\,N_{t} = \frac{{\tau D_{T} \left( {T_{w} - T_{\infty } } \right)}}{{T_{\infty } \upsilon_{f} }},\,\,\,\,Ec = \frac{{U^{2} }}{{\left( {T_{w} - T_{\infty } } \right)C_{p} }},\,\, \\ & Le = \frac{{\upsilon_{f} }}{{D_{B} }},\,\,\,\,K = \frac{{K^{*} }}{U},\,\,\,\,P_{e} = \frac{{bW_{c} }}{{D_{m} }},\,\,\,\,L_{b} = \frac{\alpha }{{D_{m} }},\,\,\,\,\varepsilon = \frac{{u_{w} }}{U} \\ \end{aligned} $$

Above $Gr_{x} = \frac{g\beta \left( {1 - C_{\infty } } \right)\left( {T_{w} - T_{\infty } } \right).x^{3} /v^{2}}{( {C_{w} - C_{\infty }) }}$ is local Grashof number and ${\text{Re}}_{x} = Ux/v$ is local Reynolds number. Also it is to be observed that the mixed convection parameter $\,\lambda > 0$ corresponds to supporting flow, whereas $\lambda < 0$ corresponds to conflicting flow. The thermophoretic characteristics for nanofluid and hybrid nanofluid are depicted in Table 1 where the numerical values for base fluid and nanoparticles are expressed in Table 2.

**Table 1:** Thermophysical properties of hybrid nanofluid^43^.

Properties	Nanofluid $\left( {Al_{2} O_{3} } \right)$	Hybrid nanofluid $\left( {Cu - Al_{2} O_{3} } \right)$
Density	$\rho_{nf} = \left( {1 - \varphi_{1} } \right)\rho_{f} + \varphi_{1} \rho_{s1}$	$\rho_{hnf} = \left\{ {\left( {1 - \varphi_{2} } \right)\left( {\left( {1 - \varphi_{1} } \right) + \varphi_{1} \frac{{\rho_{s1} }}{{\rho_{f} }}} \right) + \varphi_{2} \frac{{\rho_{s2} }}{{\rho_{f} }}} \right\}\rho_{f}$
Heat capacity	$\left( {\rho C_{p} } \right)_{nf} = \left( {1 - \varphi_{1} } \right)\left( {\rho C_{p} } \right)_{f} + \varphi_{1} \left( {\rho C_{p} } \right)_{s1}$	$$\left( {\rho C_{p} } \right)_{hnf} = \,\,\left\{ \begin{gathered} \left( {1 - \varphi_{2} } \right)\left( {\left( {1 - \varphi_{1} } \right) + \varphi_{1} \frac{{\left( {\rho C_{p} } \right)_{s1} }}{{\left( {\rho C_{p} } \right)_{f} }}} \right) \hfill \\ + \varphi_{2} \frac{{\left( {\rho C_{p} } \right)_{s2} }}{{\left( {\rho C_{p} } \right)_{f} }} \hfill \\ \end{gathered} \right\}\left( {\rho C_{p} } \right)_{f}$$
Dynamic viscosity	$\mu_{nf} = \frac{{\mu_{f} }}{{\left( {1 - \varphi_{1} } \right)^{2.5} }}$	$\mu_{hnf} = \frac{{\mu_{f} }}{{\left( {1 - \varphi_{1} } \right)^{2.5} \left( {1 - \varphi_{2} } \right)^{2.5} }}$
Thermal conductivity	$\kappa_{nf} = \frac{{\kappa_{s1} + 2\kappa_{f} - 2\varphi_{1} \left( {\kappa_{f} - \kappa_{s1} } \right)}}{{\kappa_{s1} + 2\kappa_{f} + \varphi_{1} \left( {\kappa_{f} - \kappa_{s1} } \right)}}\kappa_{f}$	$\kappa_{hnf} = \frac{{\kappa_{s2} + 2\kappa_{f} - 2\varphi_{2} \left( {\kappa_{f} - \kappa_{s2} } \right)}}{{\kappa_{s2} + 2\kappa_{f} + \varphi_{2} \left( {\kappa_{f} - \kappa_{s2} } \right)}}\kappa_{nf}$

**Table 2:** Numerical values of nanoparticles and base fluid for thermophysical properties^43^.

Properties	$\left( {Copper - Cu} \right)$ nanoparticles	$\left( {Alumina - Al_{2} O_{3} } \right)$ nanoparticles	$\left( {Water - H_{2} O} \right)$ base Fluid
$\rho \left( {{\text{kg/m}}^{3} } \right)$	8933	3970	997.1
$C_{p} \left( {{\text{J/kg}}\;{\text{K}}} \right)$	385	765	4179
$\kappa \left( {\text{W/mK}} \right)$	400	40	0.613

### Quantities of engineering interest

In the field of thermodynamics, engineers and scientists are normally interested to examine and calculate the thermal and mass transmission through fluid flow. They are also interested in determination of the resistance offered by the fluid surface and the surface of channel through which the fluid is flowing. At industrial level, liquids are mostly flowing through different parts of mechanical machinery in which the transmission rate of heat, mass and the fraction among various parts of machinery are also of more interest for engineers. In the fluid dynamics context the fraction between surface of fluid and flow channel is named as skin fraction, the heat transmission rate through fluid flow is termed as local Nusselt number and the rate of mass transmission is known as local Sherwood number. These quantities are defined mathematically as follows:

- Skin fraction
  16 $$ C_{f} = \frac{{\mu_{hnf} }}{{\rho_{f} u_{w}^{2} }}\left( {\frac{\partial u}{{\partial r}}} \right)_{r = R} $$

By employing Eq. (7) in Eq. (16) we have the following dimensionless form of skin fraction

17 $$ C_{f} {\text{Re}}_{x}^{1/2} = 4c^{1/2} \frac{1}{{\left( {1 - \varphi_{1} } \right)^{2.5} \left( {1 - \varphi_{2} } \right)^{2.5} }}f^{\prime \prime } \left( c \right) $$

- Local Nusselt Number
  18 $$ \,Nu_{x} = - \frac{{x\kappa_{hnf} }}{{k_{f} \left( {T_{w} - T_{\infty } } \right)}}\left( {\frac{\partial T}{{\partial r}}} \right)_{r = R} $$

By employing Eqn. (7) in Eqn. (18) we have the following dimensionless form of local Nusselt number

19 $$ Nu_{x} {\text{Re}}_{x}^{ - 1/2} = - 2\frac{{\kappa_{hnf} }}{{\kappa_{f} }}c^{1/2} \theta^{\prime } \left( c \right) $$

- Local Sherwood Number
  20 $$ Sh_{x} = \frac{ - x}{{\left( {C_{w} - C_{\infty } } \right)}}\left( {\frac{\partial C}{{\partial r}}} \right)_{r = R} $$

By employing Eq. (7) in Eq. (20) we have the following dimensionless form of local Sherwood number

21 $$ Sh_{x} {\text{Re}}_{x}^{1/2} = - 2c^{1/2} \phi^{\prime } \left( c \right) $$

- Local Density Number of Motile Microorganism
  22 $$ Nn_{x} = - \frac{ - x}{{n_{w} - n_{\infty } }}\left( {\frac{\partial n}{{\partial r}}} \right)_{r = R} $$

By employing Eqn. (7) in Eqn. (22) we have the following dimensionless form of local density number of motile microorganism

23 $$ Nn_{x} {\text{Re}}_{x}^{ - 1/2} = - 2c^{1/2} \xi^{\prime } \left( c \right) $$

In above equations ${\text{Re}}_{x} = \frac{Ux}{{\upsilon_{f} }}$ represents local Reynolds number.

## Method of solution

For determination the semi-analytical solution of Eqns. (9–12) using the boundary conditions stated in Eq. (14) we shall employ the semi analytical technique HAM^38,39^. Moreover, the HAM method is implemented through BVP 2.0 package. For application of this semi-analytical technique some initial guesses are required which are stated as follows:

24 $$ f_{0} \left( \eta \right) = 1 - e^{\eta } ,\,\,\,\Theta_{0} \left( \eta \right) = \frac{{\gamma_{1} }}{{1 + \gamma_{1} }}e^{ - \eta } ,\,\,\,\Phi_{0} = \frac{{\gamma_{2} }}{{1 + \gamma_{2} }}e^{ - \eta } ,\,\,\xi \left( \eta \right) = \frac{\gamma }{1 + \gamma }e^{ - \eta } $$

Such that the linear operators are expressed as

25 $$ L_{f} \left( f \right) = f^{\prime \prime \prime } - f^{\prime } ,\,\,L_{\Theta } \left( \Theta \right) = \theta^{\prime \prime } - \theta ,\,\,L_{\Phi } \left( \Phi \right) = \phi^{\prime \prime } - \phi ,\,L_{\xi } \left( \xi \right) = \xi^{\prime \prime } - \xi $$

The above linear operators in their expanded form are given as

26 $$ L_{f} \left( {e_{1} + e_{2} e^{\eta } + e_{3} e^{ - \eta } } \right) = 0,\,\,L_{\Theta } \left( {e_{4} e^{\eta } + e_{5} e^{ - \eta } } \right) = ,\,\,\,L_{\Phi } \left( {e_{6} e^{\eta } + e_{7} e^{ - \eta } } \right) = 0,\,\,L_{\xi } \left( {e_{8} e^{\eta } + e_{9} e^{ - \eta } } \right) = 0 $$

Above in Eq. (26) the expressions $e_{i} \,\,for\,\,i = 1,\,2,\,3, \ldots 8$ are constants.

Further we have

27 $$ {\rm N}_{{\overset{\lower0.5em\hbox{$\smash{\scriptscriptstyle\frown}$}}{f} }} \, \left[ {\overset{\lower0.5em\hbox{$\smash{\scriptscriptstyle\frown}$}}{f} (\eta ;\zeta ),\overset{\lower0.5em\hbox{$\smash{\scriptscriptstyle\frown}$}}{\theta } (\eta ;\zeta ),\overset{\lower0.5em\hbox{$\smash{\scriptscriptstyle\frown}$}}{\phi } (\eta ;\zeta ),\overset{\lower0.5em\hbox{$\smash{\scriptscriptstyle\frown}$}}{\xi } (\eta ;\zeta )} \right] = \frac{2}{{\left( {1 - \overset{\lower0.5em\hbox{$\smash{\scriptscriptstyle\frown}$}}{\varphi }_{1} } \right)^{2.5} \left( {1 - \overset{\lower0.5em\hbox{$\smash{\scriptscriptstyle\frown}$}}{\varphi }_{2} } \right)^{2.5} }}\left( {\eta \overset{\lower0.5em\hbox{$\smash{\scriptscriptstyle\frown}$}}{f}_{\eta \eta \eta } + \overset{\lower0.5em\hbox{$\smash{\scriptscriptstyle\frown}$}}{f}_{\eta \eta } } \right) + \left\{ {\left( {1 - \overset{\lower0.5em\hbox{$\smash{\scriptscriptstyle\frown}$}}{\varphi }_{2} } \right)\left( {\left( {1 - \overset{\lower0.5em\hbox{$\smash{\scriptscriptstyle\frown}$}}{\varphi }_{1} } \right) + \overset{\lower0.5em\hbox{$\smash{\scriptscriptstyle\frown}$}}{\varphi }_{1} \frac{{\overset{\lower0.5em\hbox{$\smash{\scriptscriptstyle\frown}$}}{\rho }_{s1} }}{{\overset{\lower0.5em\hbox{$\smash{\scriptscriptstyle\frown}$}}{\rho }_{f} }}} \right) + \overset{\lower0.5em\hbox{$\smash{\scriptscriptstyle\frown}$}}{\varphi }_{2} \frac{{\overset{\lower0.5em\hbox{$\smash{\scriptscriptstyle\frown}$}}{\rho }_{s2} }}{{\overset{\lower0.5em\hbox{$\smash{\scriptscriptstyle\frown}$}}{\rho }_{f} }}} \right\}f\overset{\lower0.5em\hbox{$\smash{\scriptscriptstyle\frown}$}}{f}_{\eta \eta } + \lambda \left( {\overset{\lower0.5em\hbox{$\smash{\scriptscriptstyle\frown}$}}{\theta } - Nr\overset{\lower0.5em\hbox{$\smash{\scriptscriptstyle\frown}$}}{\phi } - R_{b} \overset{\lower0.5em\hbox{$\smash{\scriptscriptstyle\frown}$}}{\xi } } \right) $$

28 $$ \begin{aligned} \, {\rm N}_{{\overset{\lower0.5em\hbox{$\smash{\scriptscriptstyle\frown}$}}{\theta } }} \, \left[ {\overset{\lower0.5em\hbox{$\smash{\scriptscriptstyle\frown}$}}{f} (\eta ;\zeta ),\overset{\lower0.5em\hbox{$\smash{\scriptscriptstyle\frown}$}}{\theta } (\eta ;\zeta ),\overset{\lower0.5em\hbox{$\smash{\scriptscriptstyle\frown}$}}{\phi } (\eta ;\zeta ),\overset{\lower0.5em\hbox{$\smash{\scriptscriptstyle\frown}$}}{\xi } (\eta ;\zeta )} \right] & = 2\frac{{k_{hnf} }}{{k_{f} }}\left( {\overset{\lower0.5em\hbox{$\smash{\scriptscriptstyle\frown}$}}{\theta }_{\eta } + \eta \overset{\lower0.5em\hbox{$\smash{\scriptscriptstyle\frown}$}}{\theta }_{\eta \eta } } \right) + \left\{ {\left( {1 - \overset{\lower0.5em\hbox{$\smash{\scriptscriptstyle\frown}$}}{\varphi }_{2} } \right)\left( {\left( {1 - \overset{\lower0.5em\hbox{$\smash{\scriptscriptstyle\frown}$}}{\varphi }_{1} } \right) + \overset{\lower0.5em\hbox{$\smash{\scriptscriptstyle\frown}$}}{\varphi }_{1} \frac{{\left( {\rho C_{p} } \right)_{s1} }}{{\left( {\rho C_{p} } \right)_{f} }}} \right) + \overset{\lower0.5em\hbox{$\smash{\scriptscriptstyle\frown}$}}{\varphi }_{2} \frac{{\left( {\rho C_{p} } \right)_{s2} }}{{\left( {\rho C_{p} } \right)_{f} }}} \right\}\Pr \overset{\lower0.5em\hbox{$\smash{\scriptscriptstyle\frown}$}}{f} \overset{\lower0.5em\hbox{$\smash{\scriptscriptstyle\frown}$}}{\theta }_{\eta } \\ & \quad \quad + \Pr Ec\frac{\eta }{{\left( {1 - \overset{\lower0.5em\hbox{$\smash{\scriptscriptstyle\frown}$}}{\varphi }_{1} } \right)^{2.5} \left( {1 - \overset{\lower0.5em\hbox{$\smash{\scriptscriptstyle\frown}$}}{\varphi }_{2} } \right)^{2.5} }}\left( {\overset{\lower0.5em\hbox{$\smash{\scriptscriptstyle\frown}$}}{f}_{\eta \eta } } \right)^{2} + 2\eta \Pr \left( {N_{b} \overset{\lower0.5em\hbox{$\smash{\scriptscriptstyle\frown}$}}{\theta }_{\eta } \overset{\lower0.5em\hbox{$\smash{\scriptscriptstyle\frown}$}}{\phi }_{\eta } + N_{t} \left( {\overset{\lower0.5em\hbox{$\smash{\scriptscriptstyle\frown}$}}{\theta }_{\eta } } \right)^{2} } \right) \\ \end{aligned} $$

29 $$ {\rm N}_{{\overset{\lower0.5em\hbox{$\smash{\scriptscriptstyle\frown}$}}{\phi } }} \, \left[ {\overset{\lower0.5em\hbox{$\smash{\scriptscriptstyle\frown}$}}{f} (\eta ;\zeta ),\overset{\lower0.5em\hbox{$\smash{\scriptscriptstyle\frown}$}}{\theta } (\eta ;\zeta ),\overset{\lower0.5em\hbox{$\smash{\scriptscriptstyle\frown}$}}{\phi } (\eta ;\zeta ),\overset{\lower0.5em\hbox{$\smash{\scriptscriptstyle\frown}$}}{\xi } (\eta ;\zeta )} \right] = 2\left( {\overset{\lower0.5em\hbox{$\smash{\scriptscriptstyle\frown}$}}{\phi }_{\eta } + \eta \overset{\lower0.5em\hbox{$\smash{\scriptscriptstyle\frown}$}}{\phi }_{\eta \eta } } \right) + 2\frac{{N_{t} }}{{N_{b} }}\left( {\overset{\lower0.5em\hbox{$\smash{\scriptscriptstyle\frown}$}}{\theta }_{\eta } + \eta \overset{\lower0.5em\hbox{$\smash{\scriptscriptstyle\frown}$}}{\theta }_{\eta \eta } } \right) + Le\overset{\lower0.5em\hbox{$\smash{\scriptscriptstyle\frown}$}}{\phi }_{\eta } - \frac{1}{2}LeK\overset{\lower0.5em\hbox{$\smash{\scriptscriptstyle\frown}$}}{\phi } $$

30 $$ {\rm N}_{{\overset{\lower0.5em\hbox{$\smash{\scriptscriptstyle\frown}$}}{\xi } }} \, \left[ {\overset{\lower0.5em\hbox{$\smash{\scriptscriptstyle\frown}$}}{f} (\eta ;\zeta ),\overset{\lower0.5em\hbox{$\smash{\scriptscriptstyle\frown}$}}{\theta } (\eta ;\zeta ),\overset{\lower0.5em\hbox{$\smash{\scriptscriptstyle\frown}$}}{\phi } (\eta ;\zeta ),\overset{\lower0.5em\hbox{$\smash{\scriptscriptstyle\frown}$}}{\xi } (\eta ;\zeta )} \right] = 2\left( {\overset{\lower0.5em\hbox{$\smash{\scriptscriptstyle\frown}$}}{\xi }_{\eta } + \eta \overset{\lower0.5em\hbox{$\smash{\scriptscriptstyle\frown}$}}{\xi }_{\eta \eta } } \right) + \Pr L_{b} \overset{\lower0.5em\hbox{$\smash{\scriptscriptstyle\frown}$}}{\xi } \overset{\lower0.5em\hbox{$\smash{\scriptscriptstyle\frown}$}}{f}_{\eta } - 2P_{e} \left( {2\eta \overset{\lower0.5em\hbox{$\smash{\scriptscriptstyle\frown}$}}{\xi } \phi^{\prime\prime} + \left( {\overset{\lower0.5em\hbox{$\smash{\scriptscriptstyle\frown}$}}{\xi } + \eta \overset{\lower0.5em\hbox{$\smash{\scriptscriptstyle\frown}$}}{\xi }_{\eta } } \right)\overset{\lower0.5em\hbox{$\smash{\scriptscriptstyle\frown}$}}{\phi }_{\eta } } \right) $$

It is to be noticed that the 0-order system for Eqns. (9–12) is expressed as

31 $$ (1 - \zeta )L_{{\overset{\lower0.5em\hbox{$\smash{\scriptscriptstyle\frown}$}}{f} }} \left[ {\overset{\lower0.5em\hbox{$\smash{\scriptscriptstyle\frown}$}}{f} (\eta ;\zeta ) - \overset{\lower0.5em\hbox{$\smash{\scriptscriptstyle\frown}$}}{f}_{0} (\eta )} \right] = p\hbar_{{\overset{\lower0.5em\hbox{$\smash{\scriptscriptstyle\frown}$}}{f} }} {\rm N}_{{\overset{\lower0.5em\hbox{$\smash{\scriptscriptstyle\frown}$}}{f} }} \left[ {\overset{\lower0.5em\hbox{$\smash{\scriptscriptstyle\frown}$}}{f} (\eta ;\zeta ),\overset{\lower0.5em\hbox{$\smash{\scriptscriptstyle\frown}$}}{\theta } (\eta ;\zeta ),\overset{\lower0.5em\hbox{$\smash{\scriptscriptstyle\frown}$}}{\phi } (\eta ;\zeta ),\overset{\lower0.5em\hbox{$\smash{\scriptscriptstyle\frown}$}}{\xi } (\eta ;\zeta )} \right] $$

32 $$ (1 - \zeta ) \, L_{{\overset{\lower0.5em\hbox{$\smash{\scriptscriptstyle\frown}$}}{\theta } }} \left[ {\overset{\lower0.5em\hbox{$\smash{\scriptscriptstyle\frown}$}}{\theta } (\eta ;\zeta ) - \overset{\lower0.5em\hbox{$\smash{\scriptscriptstyle\frown}$}}{\theta }_{0} (\eta )} \right] = \,\,\,p\,\hbar_{{\overset{\lower0.5em\hbox{$\smash{\scriptscriptstyle\frown}$}}{\theta } }} \,{\rm N}_{{\overset{\lower0.5em\hbox{$\smash{\scriptscriptstyle\frown}$}}{\theta } }} \,\,\left[ {\overset{\lower0.5em\hbox{$\smash{\scriptscriptstyle\frown}$}}{f} (\eta ;\zeta ),\,\,\overset{\lower0.5em\hbox{$\smash{\scriptscriptstyle\frown}$}}{\theta } (\eta ;\zeta ),\overset{\lower0.5em\hbox{$\smash{\scriptscriptstyle\frown}$}}{\phi } (\eta ,\zeta ),\overset{\lower0.5em\hbox{$\smash{\scriptscriptstyle\frown}$}}{\xi } (\eta ;\zeta )} \right] $$

33 $$ (1 - \zeta ) \, L_{{\overset{\lower0.5em\hbox{$\smash{\scriptscriptstyle\frown}$}}{\phi } }} \left[ {\overset{\lower0.5em\hbox{$\smash{\scriptscriptstyle\frown}$}}{\phi } (\eta ;\zeta )\,\,\, - \,\,\overset{\lower0.5em\hbox{$\smash{\scriptscriptstyle\frown}$}}{\phi }_{0} (\eta )} \right] = p\,\,\hbar_{{\overset{\lower0.5em\hbox{$\smash{\scriptscriptstyle\frown}$}}{\phi } }} \,\,{\rm N}_{{\overset{\lower0.5em\hbox{$\smash{\scriptscriptstyle\frown}$}}{\phi } }} \,\,\,\left[ {\overset{\lower0.5em\hbox{$\smash{\scriptscriptstyle\frown}$}}{\phi } (\eta ;\zeta )\,,\,\,\overset{\lower0.5em\hbox{$\smash{\scriptscriptstyle\frown}$}}{f} (\eta ;\zeta ),\,\,\overset{\lower0.5em\hbox{$\smash{\scriptscriptstyle\frown}$}}{\theta } \,(\eta ;\zeta ),\overset{\lower0.5em\hbox{$\smash{\scriptscriptstyle\frown}$}}{\xi } (\eta ;\zeta )} \right] $$

34 $$ (1 - \zeta ) \, L_{{\overset{\lower0.5em\hbox{$\smash{\scriptscriptstyle\frown}$}}{\xi } }} \left[ {\overset{\lower0.5em\hbox{$\smash{\scriptscriptstyle\frown}$}}{\xi } (\eta ;\zeta )\,\,\, - \,\,\overset{\lower0.5em\hbox{$\smash{\scriptscriptstyle\frown}$}}{\xi }_{0} (\eta )} \right] = p\,\,\hbar_{{\overset{\lower0.5em\hbox{$\smash{\scriptscriptstyle\frown}$}}{\xi } }} \,\,{\rm N}_{{\overset{\lower0.5em\hbox{$\smash{\scriptscriptstyle\frown}$}}{\xi } }} \,\,\,\left[ {\overset{\lower0.5em\hbox{$\smash{\scriptscriptstyle\frown}$}}{\phi } (\eta ;\zeta )\,,\,\,\overset{\lower0.5em\hbox{$\smash{\scriptscriptstyle\frown}$}}{f} (\eta ;\zeta ),\,\,\overset{\lower0.5em\hbox{$\smash{\scriptscriptstyle\frown}$}}{\theta } \,(\eta ;\zeta ),\overset{\lower0.5em\hbox{$\smash{\scriptscriptstyle\frown}$}}{\xi } (\eta ;\zeta )} \right] $$

The related boundary conditions are stated as

35 $$ \begin{aligned} & \left. {\overset{\lower0.5em\hbox{$\smash{\scriptscriptstyle\frown}$}}{f} (\eta ;\zeta )} \right|_{\eta = c} = \frac{\varepsilon }{2}c, \, \,\,\,\left. {\frac{{\partial \overset{\lower0.5em\hbox{$\smash{\scriptscriptstyle\frown}$}}{f} (\eta ;\zeta )}}{\partial \eta }} \right|_{\eta = c} = \frac{\varepsilon }{2},\left. {\,\,\,\,\overset{\lower0.5em\hbox{$\smash{\scriptscriptstyle\frown}$}}{\theta } (\eta ;\zeta )} \right|_{\eta = c} = 1,\,\,\left. {\,\,\,\,\overset{\lower0.5em\hbox{$\smash{\scriptscriptstyle\frown}$}}{\phi } (\eta ;\zeta )} \right|_{\eta = c} = 1,\,\,\,\overset{\lower0.5em\hbox{$\smash{\scriptscriptstyle\frown}$}}{\xi } \,\left. {(\eta ;\zeta )} \right|_{\eta = c} = 1, \\ & \left. {\frac{{\partial \overset{\lower0.5em\hbox{$\smash{\scriptscriptstyle\frown}$}}{f} (\eta ;\zeta )}}{\partial \eta }} \right|_{\eta \to \infty } = \frac{1}{2}\left( {1 - \varepsilon } \right),\,\,\left. {\,\,\,\,\,\,\overset{\lower0.5em\hbox{$\smash{\scriptscriptstyle\frown}$}}{\theta } (\eta ;\zeta )} \right|_{\eta \to \infty } = 0,\,\,\left. {\,\,\,\,\,\,\,\overset{\lower0.5em\hbox{$\smash{\scriptscriptstyle\frown}$}}{\phi } (\eta ;\zeta )} \right|_{\eta \to \infty } = 0,\,\,\,\,\,\,\,\,\,\,\,\overset{\lower0.5em\hbox{$\smash{\scriptscriptstyle\frown}$}}{\xi } \,\left. {(\eta ;\zeta )} \right|_{\eta \to \infty } = 0 \\ \end{aligned} $$

It is to be noticed that $\zeta \in \left[ {0,\,\,1} \right]$, so for $\zeta = 0\,\,and\,\,\zeta = 1$ we have

36 $$ \overset{\lower0.5em\hbox{$\smash{\scriptscriptstyle\frown}$}}{f} (\eta ;1) = \overset{\lower0.5em\hbox{$\smash{\scriptscriptstyle\frown}$}}{f} (\eta ),\overset{\lower0.5em\hbox{$\smash{\scriptscriptstyle\frown}$}}{\theta } (\eta ;1) = \overset{\lower0.5em\hbox{$\smash{\scriptscriptstyle\frown}$}}{\theta } (\eta ){ ,}\overset{\lower0.5em\hbox{$\smash{\scriptscriptstyle\frown}$}}{\phi } (\eta ;1) = \overset{\lower0.5em\hbox{$\smash{\scriptscriptstyle\frown}$}}{\phi } (\eta ),\,\,\overset{\lower0.5em\hbox{$\smash{\scriptscriptstyle\frown}$}}{\xi } (\eta ;1) = \overset{\lower0.5em\hbox{$\smash{\scriptscriptstyle\frown}$}}{\xi } (\eta ) $$

The expansion of Taylor’s series for $$\overset{\lower0.5em\hbox{$\smash{\scriptscriptstyle\frown}$}}{f} (\eta ;\zeta ),\,\,\overset{\lower0.5em\hbox{$\smash{\scriptscriptstyle\frown}$}}{\theta } (\eta ;\zeta ),\,\,\overset{\lower0.5em\hbox{$\smash{\scriptscriptstyle\frown}$}}{\phi } (\eta ;\zeta )\,\,and\,\,\overset{\lower0.5em\hbox{$\smash{\scriptscriptstyle\frown}$}}{\xi } (\eta ;\zeta )$$ around $$\zeta = 0$$

37 $$ \begin{aligned} & \overset{\lower0.5em\hbox{$\smash{\scriptscriptstyle\frown}$}}{f} (\eta ;\zeta ) \, = \, \overset{\lower0.5em\hbox{$\smash{\scriptscriptstyle\frown}$}}{f}_{0} (\eta ) + \sum\nolimits_{n = 1}^{\infty } {\overset{\lower0.5em\hbox{$\smash{\scriptscriptstyle\frown}$}}{f}_{n} (\eta )\zeta^{n} } \\ & \overset{\lower0.5em\hbox{$\smash{\scriptscriptstyle\frown}$}}{\theta } (\eta ;\zeta ) \, = \, \overset{\lower0.5em\hbox{$\smash{\scriptscriptstyle\frown}$}}{\theta }_{0} (\eta ) + \sum\nolimits_{n = 1}^{\infty } {\overset{\lower0.5em\hbox{$\smash{\scriptscriptstyle\frown}$}}{\theta }_{n} (\eta )\zeta^{n} } \\ & \overset{\lower0.5em\hbox{$\smash{\scriptscriptstyle\frown}$}}{\phi } (\eta ;\zeta ) \, = \, \overset{\lower0.5em\hbox{$\smash{\scriptscriptstyle\frown}$}}{\phi }_{0} (\eta ) + \sum\nolimits_{n = 1}^{\infty } {\overset{\lower0.5em\hbox{$\smash{\scriptscriptstyle\frown}$}}{\phi }_{n} (\eta )\zeta^{n} } \\ & \overset{\lower0.5em\hbox{$\smash{\scriptscriptstyle\frown}$}}{\xi } (\eta ;\zeta ) = \, \overset{\lower0.5em\hbox{$\smash{\scriptscriptstyle\frown}$}}{\xi }_{0} (\eta ) + \sum\nolimits_{n = 1}^{\infty } {\overset{\lower0.5em\hbox{$\smash{\scriptscriptstyle\frown}$}}{\xi }_{n} (\eta )\zeta^{n} } \\ \end{aligned} $$

38 $$ \begin{aligned} & \overset{\lower0.5em\hbox{$\smash{\scriptscriptstyle\frown}$}}{f}_{n} (\eta ) \, = \left. {\frac{1}{n!}\frac{{\partial \overset{\lower0.5em\hbox{$\smash{\scriptscriptstyle\frown}$}}{f} (\eta ;\zeta )}}{\partial \eta }} \right|_{p = 0} ,\overset{\lower0.5em\hbox{$\smash{\scriptscriptstyle\frown}$}}{\theta }_{n} (\eta ) \, = \left. {\frac{1}{n!}\frac{{\partial \overset{\lower0.5em\hbox{$\smash{\scriptscriptstyle\frown}$}}{\theta } (\eta ;\zeta )}}{\partial \eta }} \right|_{p = 0} , \\ & \overset{\lower0.5em\hbox{$\smash{\scriptscriptstyle\frown}$}}{\phi }_{n} (\eta ) \, = \left. {\frac{1}{n!}\frac{{\partial \overset{\lower0.5em\hbox{$\smash{\scriptscriptstyle\frown}$}}{\phi } (\eta ;\zeta )}}{\partial \eta }} \right|_{p = 0} ,\,\,\overset{\lower0.5em\hbox{$\smash{\scriptscriptstyle\frown}$}}{\xi }_{n} (\eta ) \, = \left. {\frac{1}{n!}\frac{{\partial \overset{\lower0.5em\hbox{$\smash{\scriptscriptstyle\frown}$}}{\xi } (\eta ;\zeta )}}{\partial \eta }} \right|_{p = 0} \\ \end{aligned} $$

With boundary conditions as follows

39 $$ \begin{array}{*{20}l} {\overset{\lower0.5em\hbox{$\smash{\scriptscriptstyle\frown}$}}{f} \left( c \right) = \frac{\varepsilon }{2}c,\,\,\,\,\overset{\lower0.5em\hbox{$\smash{\scriptscriptstyle\frown}$}}{f}^{\prime } \left( c \right) = \frac{\varepsilon }{2},\,\,\,\,\overset{\lower0.5em\hbox{$\smash{\scriptscriptstyle\frown}$}}{\theta } \left( c \right) = 1,\,\,\,\,\overset{\lower0.5em\hbox{$\smash{\scriptscriptstyle\frown}$}}{\phi } \left( c \right) = 1,\,\,\,\,\overset{\lower0.5em\hbox{$\smash{\scriptscriptstyle\frown}$}}{\xi } \left( c \right) = 1\,\,} \hfill \\ {\overset{\lower0.5em\hbox{$\smash{\scriptscriptstyle\frown}$}}{f}^{\prime } \left( \infty \right) = \frac{1}{2}\left( {1 - \varepsilon } \right),\,\,\,\,\,\,\overset{\lower0.5em\hbox{$\smash{\scriptscriptstyle\frown}$}}{\theta } \left( \infty \right) = 0,\,\,\,\,\,\,\overset{\lower0.5em\hbox{$\smash{\scriptscriptstyle\frown}$}}{\phi } \left( \infty \right) = 0,\,\,\,\,\,\overset{\lower0.5em\hbox{$\smash{\scriptscriptstyle\frown}$}}{\xi } \left( \infty \right) = 0} \hfill \\ \end{array} $$

Next we have

40 $$ \Re_{n}^{{\overset{\lower0.5em\hbox{$\smash{\scriptscriptstyle\frown}$}}{f} }} \left( \eta \right) = \frac{2}{{\left( {1 - \overset{\lower0.5em\hbox{$\smash{\scriptscriptstyle\frown}$}}{\varphi }_{1} } \right)^{2.5} \left( {1 - \overset{\lower0.5em\hbox{$\smash{\scriptscriptstyle\frown}$}}{\varphi }_{2} } \right)^{2.5} }}\left( {\eta \overset{\lower0.5em\hbox{$\smash{\scriptscriptstyle\frown}$}}{f}_{n - 1}^{\prime \prime \prime } + \overset{\lower0.5em\hbox{$\smash{\scriptscriptstyle\frown}$}}{f}_{n - 1}^{\prime \prime } } \right) + \left\{ {\left( {1 - \overset{\lower0.5em\hbox{$\smash{\scriptscriptstyle\frown}$}}{\varphi }_{2} } \right)\left( {\left( {1 - \overset{\lower0.5em\hbox{$\smash{\scriptscriptstyle\frown}$}}{\varphi }_{1} } \right) + \overset{\lower0.5em\hbox{$\smash{\scriptscriptstyle\frown}$}}{\varphi }_{1} \frac{{\overset{\lower0.5em\hbox{$\smash{\scriptscriptstyle\frown}$}}{\rho }_{s1} }}{{\overset{\lower0.5em\hbox{$\smash{\scriptscriptstyle\frown}$}}{\rho }_{f} }}} \right) + \overset{\lower0.5em\hbox{$\smash{\scriptscriptstyle\frown}$}}{\varphi }_{2} \frac{{\overset{\lower0.5em\hbox{$\smash{\scriptscriptstyle\frown}$}}{\rho }_{s2} }}{{\overset{\lower0.5em\hbox{$\smash{\scriptscriptstyle\frown}$}}{\rho }_{f} }}} \right\}\sum\limits_{j = 0}^{w - 1} {\overset{\lower0.5em\hbox{$\smash{\scriptscriptstyle\frown}$}}{f}_{w - 1 - j} \overset{\lower0.5em\hbox{$\smash{\scriptscriptstyle\frown}$}}{f}_{j}^{\prime \prime } } + \lambda \left( {\overset{\lower0.5em\hbox{$\smash{\scriptscriptstyle\frown}$}}{\theta } - Nr\overset{\lower0.5em\hbox{$\smash{\scriptscriptstyle\frown}$}}{\phi } - R_{b} \overset{\lower0.5em\hbox{$\smash{\scriptscriptstyle\frown}$}}{\xi } } \right) $$

41 $$ \Re \,_{n}^{{\overset{\lower0.5em\hbox{$\smash{\scriptscriptstyle\frown}$}}{\theta } }} \,\left( \eta \right) = 2\frac{{k_{hnf} }}{{k_{f} }}\left( {\overset{\lower0.5em\hbox{$\smash{\scriptscriptstyle\frown}$}}{\theta }_{n - 1} + \eta \overset{\lower0.5em\hbox{$\smash{\scriptscriptstyle\frown}$}}{\theta }_{n - 1} } \right) + \left\{ {\left( {1 - \overset{\lower0.5em\hbox{$\smash{\scriptscriptstyle\frown}$}}{\varphi }_{2} } \right)\left( {\left( {1 - \overset{\lower0.5em\hbox{$\smash{\scriptscriptstyle\frown}$}}{\varphi }_{1} } \right) + \overset{\lower0.5em\hbox{$\smash{\scriptscriptstyle\frown}$}}{\varphi }_{1} \frac{{\left( {\rho C_{p} } \right)_{s1} }}{{\left( {\rho C_{p} } \right)_{f} }}} \right) + \overset{\lower0.5em\hbox{$\smash{\scriptscriptstyle\frown}$}}{\varphi }_{2} \frac{{\left( {\rho C_{p} } \right)_{s2} }}{{\left( {\rho C_{p} } \right)_{f} }}} \right\}\Pr \sum\limits_{j = 0}^{w - 1} {\overset{\lower0.5em\hbox{$\smash{\scriptscriptstyle\frown}$}}{f}_{w - 1 - j} } \overset{\lower0.5em\hbox{$\smash{\scriptscriptstyle\frown}$}}{\theta }_{n - 1} + \Pr Ec\frac{\eta }{{\left( {1 - \overset{\lower0.5em\hbox{$\smash{\scriptscriptstyle\frown}$}}{\varphi }_{1} } \right)^{2.5} \left( {1 - \overset{\lower0.5em\hbox{$\smash{\scriptscriptstyle\frown}$}}{\varphi }_{2} } \right)^{2.5} }}\left( {\overset{\lower0.5em\hbox{$\smash{\scriptscriptstyle\frown}$}}{f}_{n - 1} } \right)^{2} + 2\eta \Pr \left( {N_{b} \overset{\lower0.5em\hbox{$\smash{\scriptscriptstyle\frown}$}}{\theta }_{n - 1} \overset{\lower0.5em\hbox{$\smash{\scriptscriptstyle\frown}$}}{\phi }_{n - 1} + N_{t} \left( {\overset{\lower0.5em\hbox{$\smash{\scriptscriptstyle\frown}$}}{\theta }_{n - 1} } \right)^{2} } \right) $$

42 $$ \Re_{n}^{{\overset{\lower0.5em\hbox{$\smash{\scriptscriptstyle\frown}$}}{\phi } }} (\eta ) = 2\left( {\overset{\lower0.5em\hbox{$\smash{\scriptscriptstyle\frown}$}}{\phi }_{n - 1} + \eta \overset{\lower0.5em\hbox{$\smash{\scriptscriptstyle\frown}$}}{\phi }_{n - 1} } \right) + 2\frac{{N_{t} }}{{N_{b} }}\left( {\overset{\lower0.5em\hbox{$\smash{\scriptscriptstyle\frown}$}}{\theta }_{n - 1} + \eta \overset{\lower0.5em\hbox{$\smash{\scriptscriptstyle\frown}$}}{\theta }_{n - 1} } \right) + Le\overset{\lower0.5em\hbox{$\smash{\scriptscriptstyle\frown}$}}{\phi }_{n - 1} - \frac{1}{2}LeK\sum\limits_{j = 0}^{w - 1} {\phi_{w - 1 - j} } $$

43 $$ \Re_{n}^{{\overset{\lower0.5em\hbox{$\smash{\scriptscriptstyle\frown}$}}{\xi } }} (\eta ) = \, 2\left( {\overset{\lower0.5em\hbox{$\smash{\scriptscriptstyle\frown}$}}{\xi }_{n - 1} + \eta \overset{\lower0.5em\hbox{$\smash{\scriptscriptstyle\frown}$}}{\xi }_{n - 1} } \right) + \Pr L_{b} \sum\limits_{j = 0}^{w - 1} {\overset{\lower0.5em\hbox{$\smash{\scriptscriptstyle\frown}$}}{\xi }_{w - 1 - j} } \overset{\lower0.5em\hbox{$\smash{\scriptscriptstyle\frown}$}}{f}_{n - 1} - 2P_{e} \left( {2\eta \sum\limits_{j = 0}^{w - 1} {\overset{\lower0.5em\hbox{$\smash{\scriptscriptstyle\frown}$}}{\xi }_{w - 1 - j} } \overset{\lower0.5em\hbox{$\smash{\scriptscriptstyle\frown}$}}{\xi } \phi_{n - 1}^{\prime \prime } + \left( {\sum\limits_{j = 0}^{w - 1} {\overset{\lower0.5em\hbox{$\smash{\scriptscriptstyle\frown}$}}{\xi }_{w - 1 - j} } \overset{\lower0.5em\hbox{$\smash{\scriptscriptstyle\frown}$}}{\xi } + \eta \overset{\lower0.5em\hbox{$\smash{\scriptscriptstyle\frown}$}}{\xi }_{n - 1} } \right)\overset{\lower0.5em\hbox{$\smash{\scriptscriptstyle\frown}$}}{\phi }_{n - 1} } \right) $$

Moreover, we have

44 $$ \chi_{n} = \left\{ {\begin{array}{*{20}l} {0,} \hfill & {{\text{if }}\zeta \le {1}} \hfill \\ {1,} \hfill & {{\text{if }}\zeta > {1}{\text{.}}} \hfill \\ \end{array} } \right. $$

## Results and discussion

**Figure 2:**
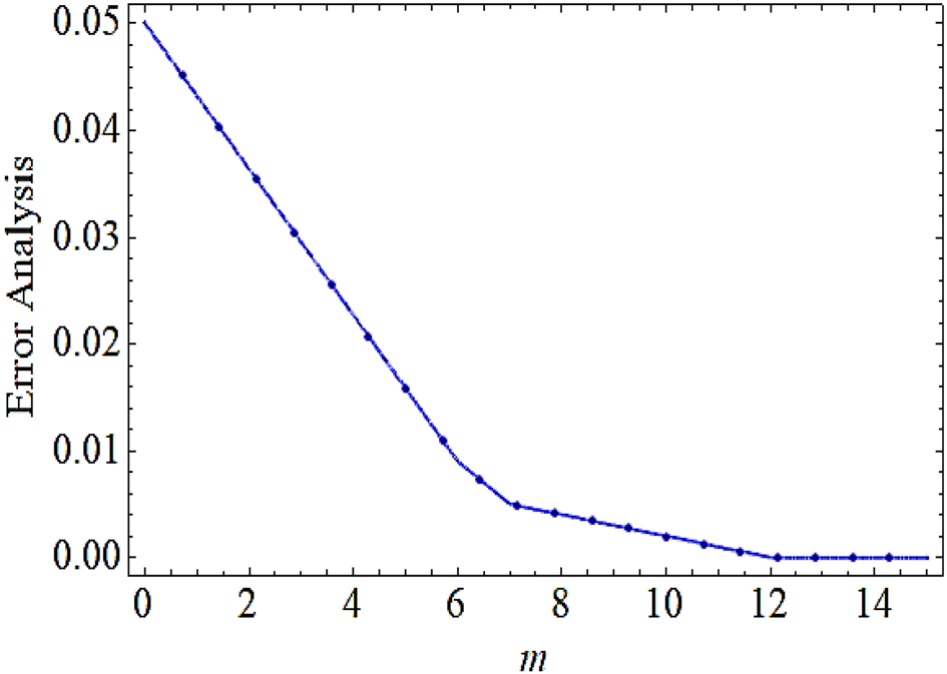
Total square residual error for modelled problem.

In this investigation the thermal analysis for bio-convective hybrid nanofluid flowing upon a thin horizontally moving needle is carried out. The hybrid nanoparticles comprising of copper and alumina are considered for current flow problem. Mathematically the flow problem is formulated by employing the famous Buongiorno’s model that will also investigate the consequences of thermophoretic forces and Brownian motion upon flow system. HAM is used to determine solution of set of dimensionless equations. The impact of various physical parameters upon flow, thermal, concentration characteristics and density of motile microorganism with the help of graphical view have discussed. The problem geometry is depicted in Fig. 1. The total square residual error displayed in Fig. 2 using the BVP 2.0 package of HAM. The strong convergence obtained up to the 15 iterations order.

### Flow characteristics $f^{\prime } \left( \eta \right)$

**Figure 3:**
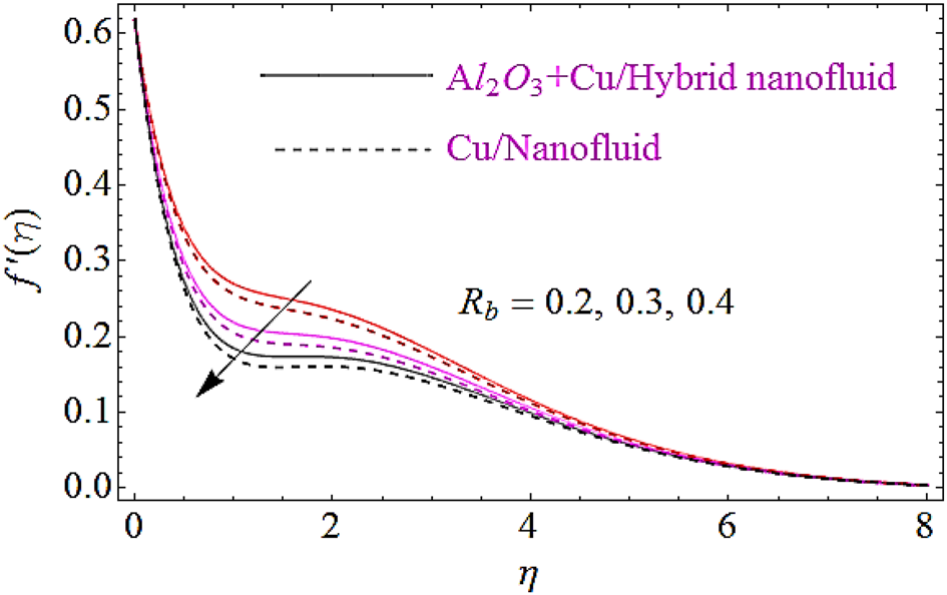
Flow characteristics for dissimilar values of $R_{b}$.

**Figure 4:**
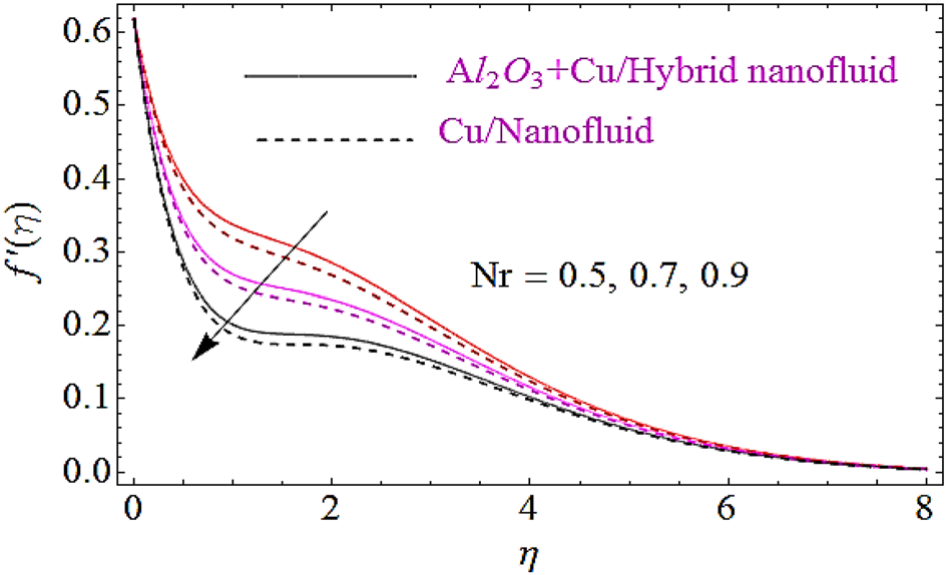
Flow characteristics for dissimilar values of $Nr$.

**Figure 5:**
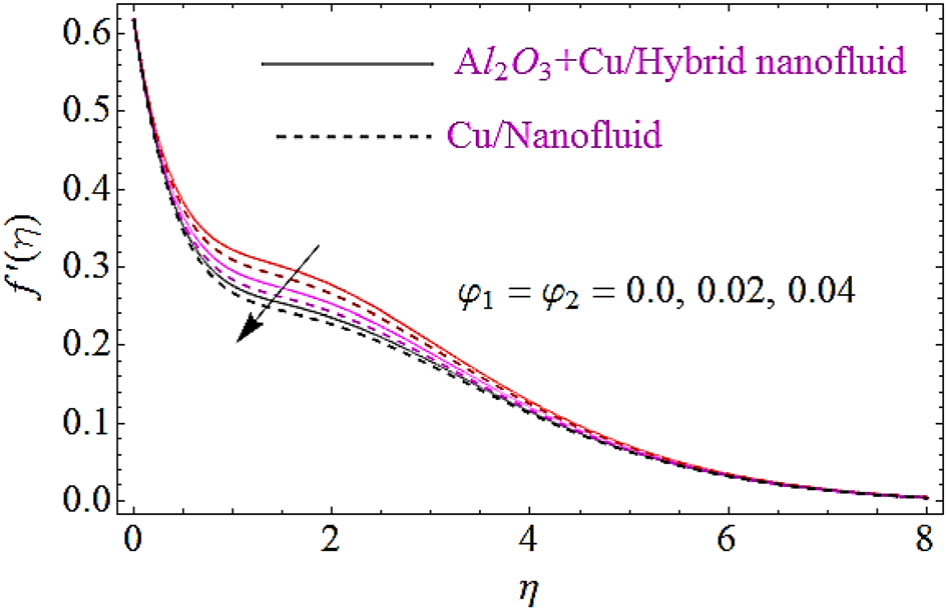
Flow characteristics for dissimilar values of $\varphi_{1} = \varphi_{2}$.

In this subsection the impact of the emerging parameters such as bioconvection Rayleigh number $\left( {R_{b} } \right)$, buoyancy ratio parameter $\left( {Nr} \right)$ and volumetric fraction $\varphi_{1} ,\,\varphi_{2}$ for nanoparticles upon flow profiles of nanofluid will be discussed, as depicted in Figs. 3, 4, 5. From Fig. 3 it is perceived that flow declines with augmentation in $R_{b}$. Physically a growth in the values of $R_{b}$ offers a resistance to the upward motion of nanoparticles which declines the flow characteristics of fluid. The impact $Nr$ on flow is describes in Fig. 4. Physically it can be interpreted as an increase in $Nr$ moves the nanfluid towards the needle’s surface. Additionally an augmentation in the inverse bouncy exaggerated by volume fraction of nanoparticle at free stream and results in reduction of the flow distribution and wideness of momentum boundary layer. This ultimately declines the flow of fluid. The impact of volumetric fractions $\varphi_{1} ,\,\varphi_{2}$ of nanoparticles upon flow characteristics is exposed in Fig. 5. Since with growth in the values of $\varphi_{1}$ or $\,\varphi_{2}$,there is a corresponding increase in the viscous nature of nanofluid, due to this physical phenomenon flow of fluid declines.

### Thermal characteristics $\theta \left( \eta \right)$

**Figure 6:**
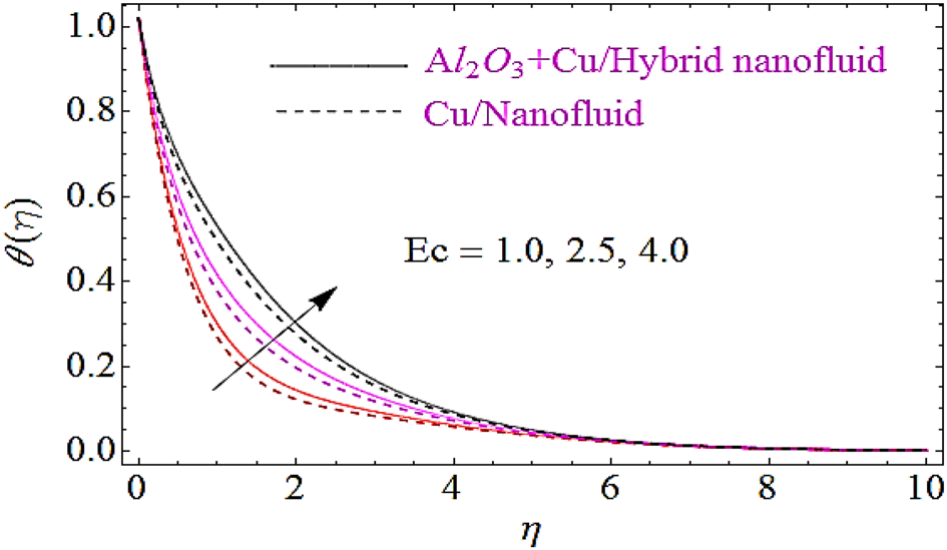
Thermal characteristics for dissimilar values of $Ec$.

**Figure 7:**
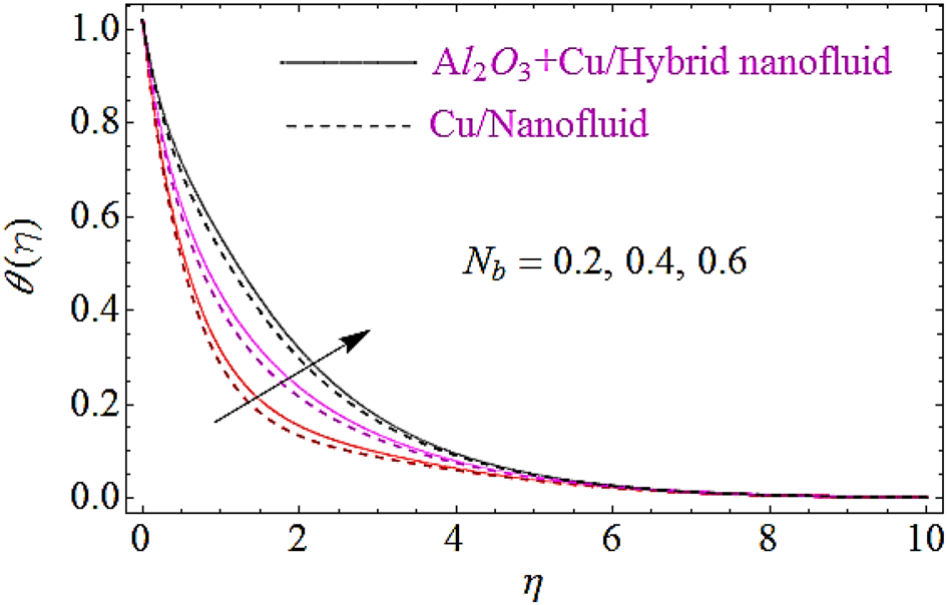
Flow characteristics for different values of $N_{b}$.

**Figure 8:**
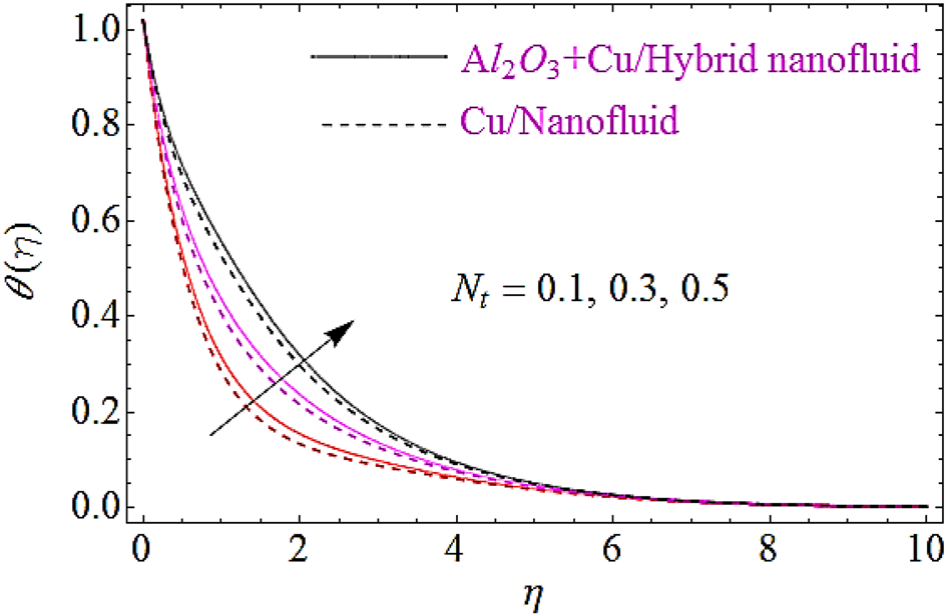
Thermal characteristics for different values of $N_{t}$.

**Figure 9:**
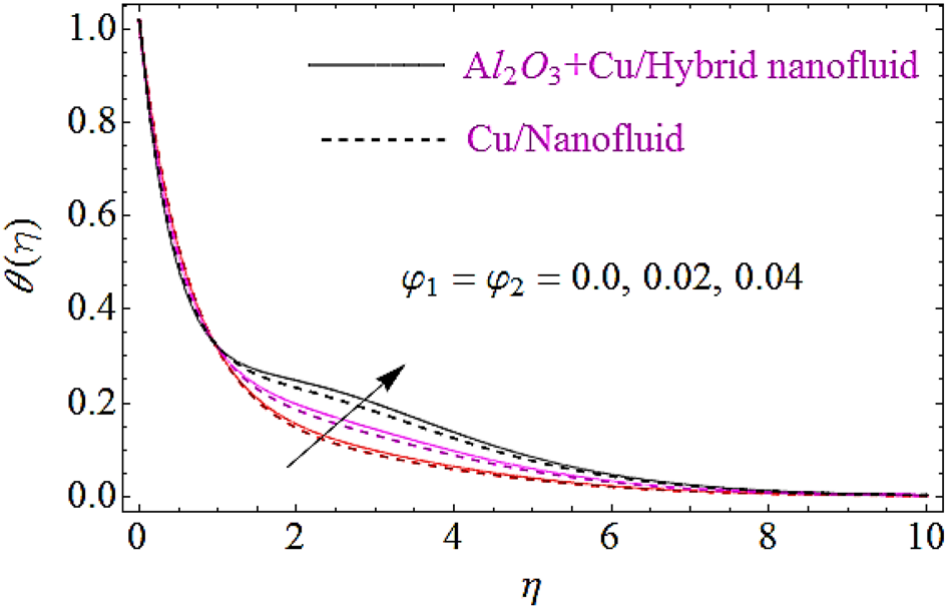
Thermal characteristics for different values of $\varphi_{1} = \varphi_{2}$.

The impact of Eckert number $\left( {Ec} \right)$, Brownian motion parameter $\left( {N_{b} } \right)$, Thermophoretic parameter $\left( {N_{t} } \right)$ and volumetric fractions $\left( {\varphi_{1} = \,\varphi_{2} } \right)$ of nanoparticles upon thermal characteristics is discussed in Figs. 6, 7, 8, 9. Figure 6 depicts the impact of Eckert number upon $\theta \left( \eta \right)$. Since growth in $Er$ enhances the transportation energy due to which thermal boundary layer of nanoparticles increases. Hence thermal characteristics grow up due to increase in Eckert number. The growth in $N_{b}$ results in augmentation of nanoparticles random collision which grows up the thermal boundary layer, because during this physical phenomenon the kinetic energy of nanoparticles is converted to heat energy. Hence increase in $N_{b}$ corresponds to a growth in thermal characteristics as depicted in Fig. 7. Similarly increase in thermophoretic parameter results in an increase in temperature gradient of nanoparticles. Actually, for larger value of $N_{t}$ there will be maximum temperature gradient in the flow system that leads to a maximum heat transfer as shown in Fig. 8. The rise in the volume fractions of alumina $\left( {Al_{2} O_{3} } \right)$ or copper $\left( {Cu} \right)$ nanoparticles results in an augmentation in density of fluid. During this physical phenomenon the thermal boundary layer of nanofluid enhances as depicted in Fig. 9.

### Concentration characteristics $\phi \left( \eta \right)$

**Figure 10:**
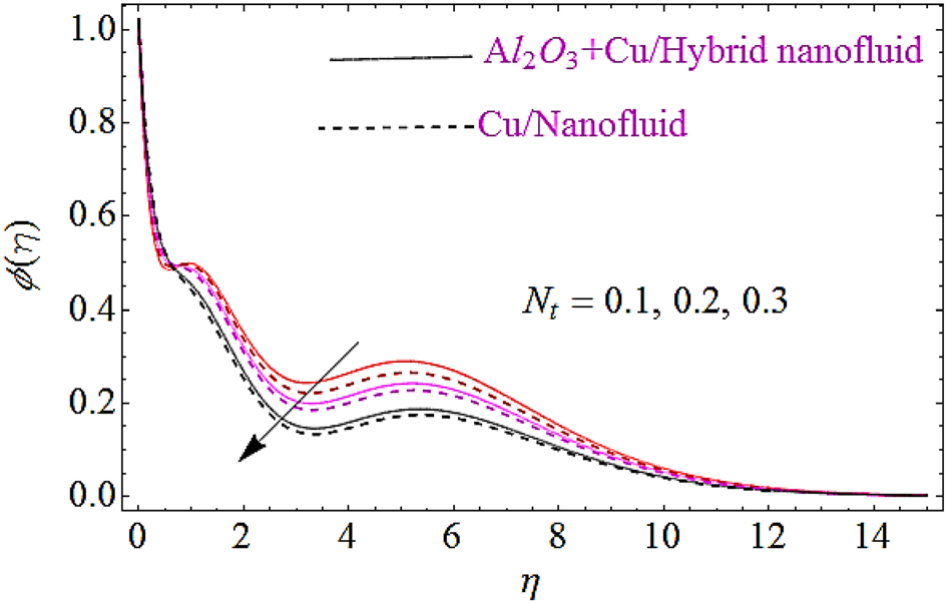
Concentration characteristics for different values of $N_{t}$.

**Figure 11:**
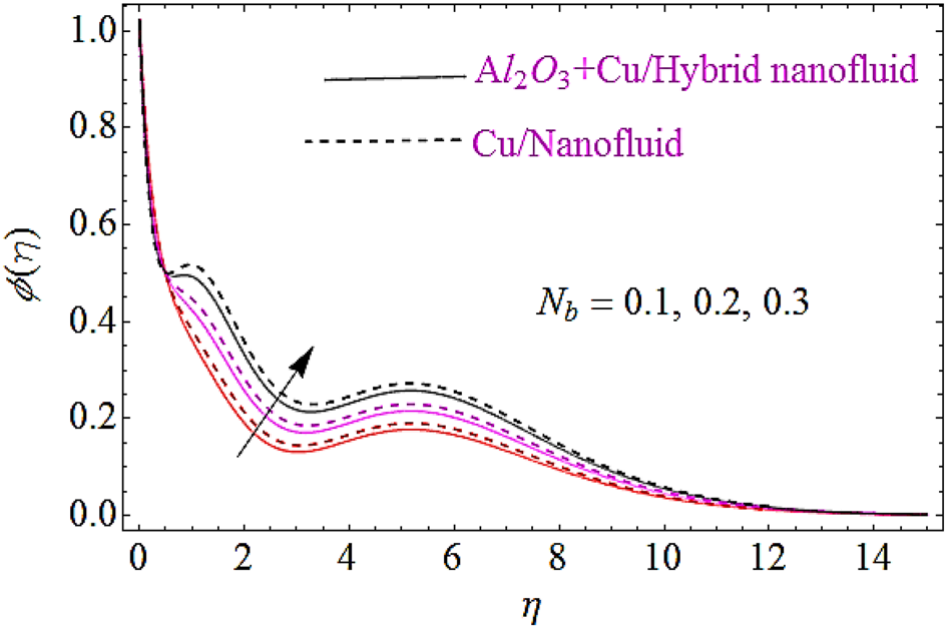
Concentration characteristics for different values of $N_{b}$.

**Figure 12:**
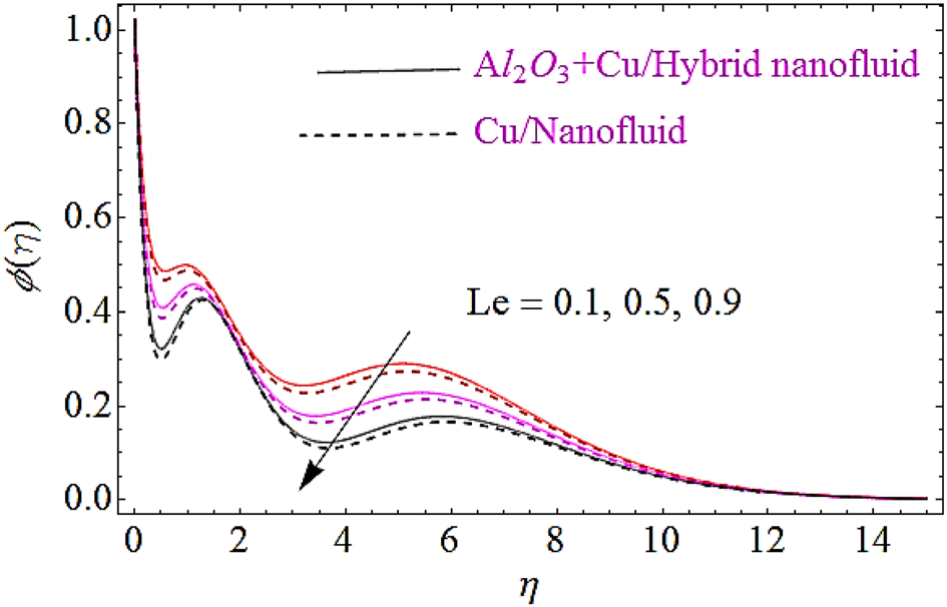
Concentration characteristics for different values of $Le$.

In this subsection impact of thermophoretic parameter $\left( {N_{t} } \right)$, Brownian motion parameter $\left( {N_{b} } \right)$ and Lewis number $\left( {Le} \right)$ will be discussed, as shown in Figs. 10, 11, 12. From Fig. 10 it is observed that with a growth in $N_{t}$ the thermal conductivity of nanofluid grows up and also infiltrates deeper in the nanoparticles and finally declines the thickness of concertation boundary layer. Hence increase in thermophoresis parameter corresponds to reduction in concentration characteristics. Figure 11 depicts impact of Brownian motion parameter upon concentration of nanofluid. Since the mass transfer rate declines with augmentation in Brownian motion parameter that declines the concentration boundary thickness of naofluid. Hence augmentation in Brownian motion results in reduction of concentration characteristics as shown in Fig. 11. Moreover, augmentation in Lewis number reduces the mass flow that further weakens the concentration boundary layer. Hence increase in Lewis number declines the concentration characteristics of nanofluid as depicted in Fig. 12.

### Motile microorganism characteristics $\xi \left( \eta \right)$

**Figure 13:**
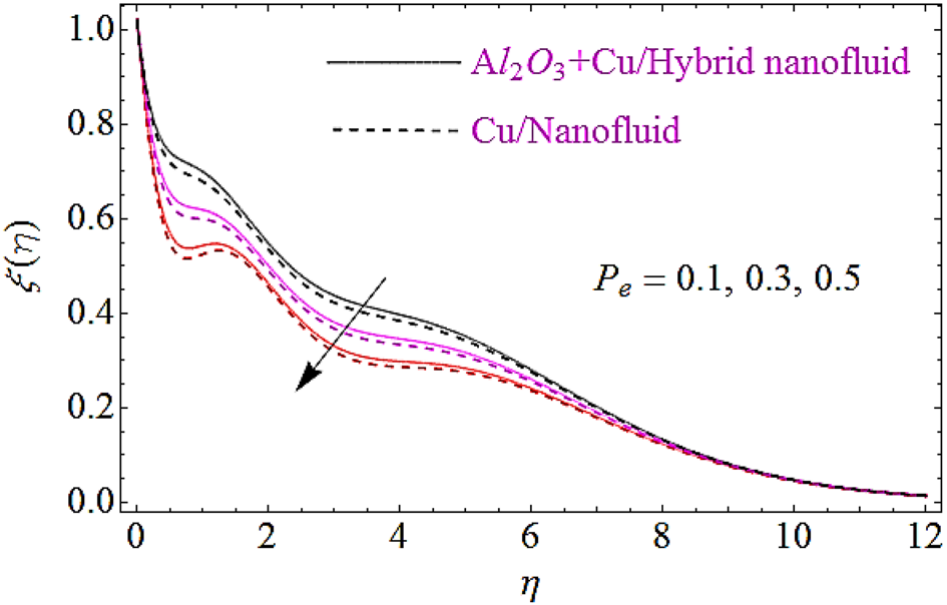
Motile microorganism characteristics for different values of $P_{e}$.

**Figure 14:**
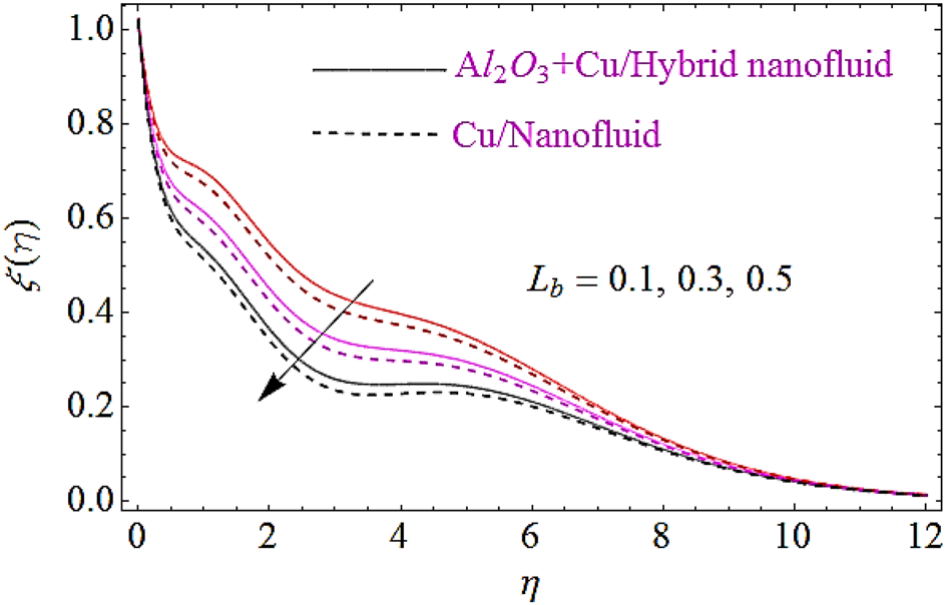
Motile microorganism characteristics for different values of $L_{b}$.

The impact of Peclet number $\left( {P_{e} } \right)$ and bioconvection Lewis number $\left( {L_{b} } \right)$ upon motile microorganism characteristics is depicted in Figs. 13, 14. It can be noticed from these figure that higher variations in $L_{b}$ and $P_{e}$ results in decline of dimensionless microorganism of nanofluid. Actually an augmentation in the values of $P_{e}$, $L_{b}$ leads to a less spread of microorganism and results a decline of motile boundary layer thickness of nanofluid. Physically the motile density declines with increasing numerical values of $P_{e}$ or $L_{b}$ that ultimately results in the reduction of motile microorganism of nanofluid.

### Flow chart of HAM and comparison

**Figure 15:**
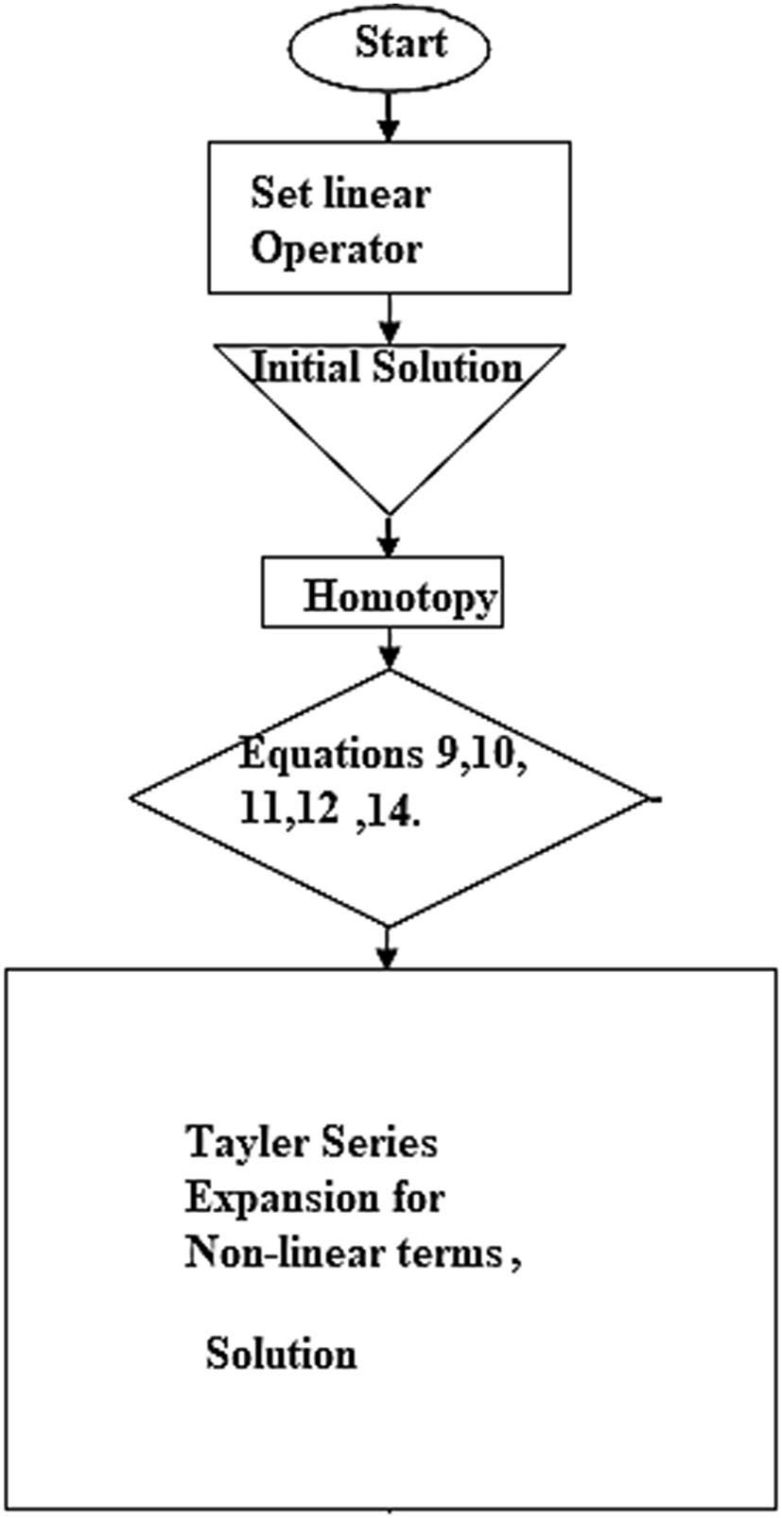
Flow chart for HAM technique.

**Figure 16:**
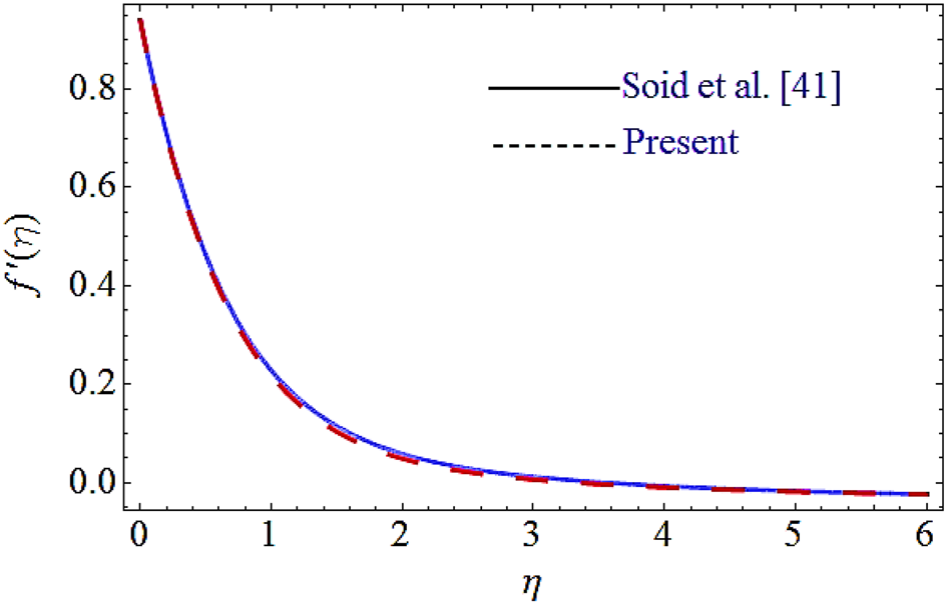
Comparison of current results with Ref.^41^ for velocity profile.

**Figure 17:**
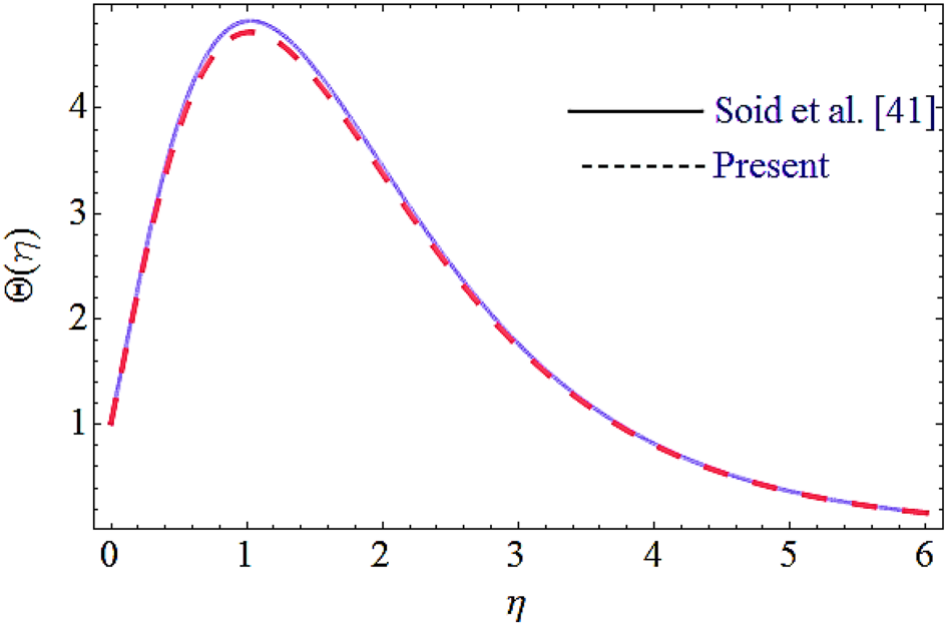
Comparison of current results with Ref.^41^ for thermal profile.

The flow chart of the HAM method is added and depicted in Fig. 15. Moreover, the comparison of the present results with the published work is displayed in Figs. 16, 17 by considering common parameters. From these figures a closed agreement of the present and published results has been observed, which shows the authentication of the obtained results.

### Table discussion

The numerical outputs of the present study are displayed in Tables 1, 2, 3, 4, 5. The thermophysical characteristics are displayed in the Table 1. The numerical values of thermophysical properties for solid nanoparticles and base liquid are presented in Table 2. The focus has been given to the nanoparticle’s volume fraction and the percentage enhancement in skin friction coefficient and heat transfer rate for both $Cu$ nanofluid and $Cu + Al_{2} O_{3}$ hybrid nanofluid as described in Tables 3, 4. From Table 3 it is observed that the increase in the magnitude of volume fraction of Cu-nanoparticles volume fraction $\varphi_{1}$ from 0.0 to 0.01 and 0.0 to 0.02 enhances the skin friction coefficient from 2.667% to 5.4005% respectively. While for the same variations in values of $Cu + Al_{2} O_{3}$-nanoparticles volume fraction $\varphi_{1} = \varphi_{2}$ enhances the skin friction coefficient from 6.7% to13.7%, showing that the hybrid nanofluid increases the skin friction more rapidly and efficiently as compared to other traditional fluids. From Table 4 it is perceived that the percentage increase in the heat transfer rate for variations in $Cu$-nanoparticles volume fraction $\varphi_{1}$ from 0.0 to 0.01 and 0.0 to 0.02 are respectively 3.27409% and 6.637%. On the other hand for the same variations in values of $Cu + Al_{2} O_{3}$-nanoparticles volume fraction $\varphi_{1} = \varphi_{2}$ the heat transfer rate is observed as 3.324% and 6.742% respectively. This shows that the hybrid nanofluid enhancing the heat transfer rate more efficiently as compared to the other traditional fluids. The comparison of the present study with the existing literature is carried out and presented in Table 5. A closed agreement has been observed in both results by varying the thickness of the needle.

**Table 3:** Percentage growth in the skin friction due to solid nanoparticle volume fraction, when $Nr = Nb = Nt = 0.1,\Pr = 6.2,\varepsilon = 1.1,c = 0.02.$

$\varphi_{1} = \varphi_{2}$	$4c^{1/2} (1 - \varphi_{1} )^{ - 2.5} f^{\prime \prime } (c)$ $\left( {Cu} \right)$	% Increase	$4c^{1/2} (1 - \varphi_{1} )^{ - 2.5} (1 - \varphi_{2} )^{ - 2.5} f^{\prime \prime } (c)$ $\left( {Al_{2} O_{3} + Cu} \right)$	% Increase
0.0	0.376002		0.376002	
0.01	0.386032	2.667%	0.401483	6.7%
0.02	0.396308	5.4005%	0.427769	13.7%

**Table 4:** Percentage increase in the rate of heat transfer due to solid nanoparticle volume fraction, when $Nr = Nb = Nt = 0.1,\Pr = 6.2,\varepsilon = 1.1,c = 0.02$.

$\varphi_{1} = \varphi_{2}$	$- 2\kappa_{nf} {/}\kappa_{f} \,c^{1/2} \theta^{\prime } \left( c \right)$ $\left( {Cu} \right)$	% Increase	$- 2\kappa_{hnf} {/}\kappa_{f} \,c^{1/2} \theta^{\prime } \left( c \right)$ $\left( {Al_{2} O_{3} + Cu} \right)$	% Increase
0.0	1.83318		1.83318	
0.01	1.89320	3.27409%	1.89413	3.324%
0.02	1.95485	6.6371%	1.95679	6.742%

**Table 5:** Comparison with the existing literature^41^ using only common parameters $\Pr = 6.2$.

$c$	$4c^{1/2} f^{\prime \prime } (c)$ Soid and Pop^41^	$4c^{1/2} f^{\prime \prime } (c)$ present	$- 2c^{1/2} \theta^{\prime}\left( c \right)$ Soid and Pop^41^	$- 2c^{1/2} \theta^{\prime}\left( c \right)$ Present	$- 2c^{1/2} \phi^{\prime } \left( c \right)$	$- 2c^{1/2} \xi^{\prime } \left( c \right)$
0.1	0.864546	0.864657	1.53162	1.531735	1.70480	1.43433
0.2	0.970583	0.970694	1.89531	1.895422	1.18017	1.04621
0.3	1.07510	1.075212	2.48240	2.482513	0.788956	0.742106

## Conclusion

In this investigation the thermal analysis for bio-convective hybrid nanofluid flowing upon a thin horizontally moving needle is carried out. The hybrid nanoparticles comprising of copper and alumina are considered for current flow problem. Mathematically the flow problem is formulated by employing the famous Buongiorno’s model that will also investigate the consequences of thermophoretic forces and Brownian motion upon flow system. HAM is used to determine solution of set of dimensionless equations. The impact of various physical parameters upon flow, thermal, concentration characteristics and density of motile microorganism with the help of graphical view have discussed. After detail study of the work the following points are highlighted (Supplementary Files):

- Growth in the values of bioconvection Rayleigh number offers a resistance to the upward motion of nanoparticles due to which flow of fluid declines.
- Increase in buoyancy ratio parameter moves the nanofluid towards the surface of the needle and results in reduction of the flow distribution
- An augmentation in the values of volume fractions of nanoparticles also reduces velocity profile.
- Rise in values of Eckert number enhances the transportation energy due to which thermal boundary layer of nanoparticles increases, hence temperature grows due to increase in Eckert number.
- The growth in Brownian motion results in augmentation of nanoparticles random collision which grows up the thermal boundary layer that ultimately rises the temperature. On the other hand concentration of nanofluid reduces during this physical phenomenon.
- Increase in thermophoretic parameter results in increase of temperature gradient of nanoparticles and hence maximum heat will transfer. Moreover, concentration of nanofluid also enhances during this phenomenon.
- The rise in volume fractions of alumina $\left( {Al_{2} O_{3} } \right)$ or copper $\left( {Cu} \right)$ nanoparticles results in augmentation in thermal boundary layer of nanofluid.
- Augmentation in Lewis number reduces the mass flow that further weakens the concentration boundary layer. Hence increase in Lewis number declines the concentration characteristics of nanofluid.
- Augmentation in Peclet and bioconvection Lewis numbers has an adverse impact upon motile microorganism profile, as it reduces due to increase in above-mentioned numbers.
- The increase in the magnitude of volume fraction of Cu-nanoparticles from 0.0 to 0.01 and 0.0 to 0.02 enhances the skin friction coefficient from 2.667% to 5.4005%. While the skin friction coefficient enhances from 6.7% to13.7% for the same variations in values of volume fraction of $Cu + Al_{2} O_{3}$-nanoparticles, showing that the hybrid nanofluid increases the skin friction more rapidly and efficiently as compared to other traditional fluids.
- The percentage increase in the heat transfer rate for variations in volume fraction of $Cu$-nanoparticles from 0.0 to 0.01 and 0.0 to 0.02 are respectively 3.27409% and 6.637%. On the other hand for the same variations in values of volume fraction of $Cu + Al_{2} O_{3}$-nanoparticles the heat transfer rate is observed as 3.324% and 6.742% respectively. This shows that the hybrid nanofluid enhancing the heat transfer rate more efficiently as compared to the other traditional fluids.
- A comparison between current results and the results available in literature has also been carried out both graphically and numerically in tabular form. A fine agreement has established between both the results.
- In future, the above investigation can also be extended by incorporating the effects of variable thermal conductivity, variable viscosity. Moreover, micropolar fluid can also be considered in the mathematical model of current investigation.

## Supplementary Information

Supplementary Information 1.

Supplementary Information 2.

Supplementary Information 3.

Supplementary Information 4.

Supplementary Information 5.

Supplementary Information 6.

## List of symbols

$$T_{w}$$: Wall temperature $$\left( {\text{K}} \right)$$
$T_{\infty }$: Fluid temperature at free stream $\left( {\text{K}} \right)$
$$C_{w}$$: Wall concentration
$C_{\infty }$: Fluid concentration at free stream
$n_{w}$: Microorganism at wall
$n_{\infty }$: Microorganism at free stream
$D_{B}$: Brownian diffusion coefficient $\left( {{\text{m}}^{2} {\text{/s}}} \right)$
$D_{T}$: Thermophoresis diffusion coefficient $\left( {{\text{m}}^{2} {\text{/s}}} \right)$
$N_{b}$: Brownian diffusion parameter
$Nr$: Buoyancy ratio parameter
$$N_{t}$$: Thermophoretic parameter
$K^{*}$: Rate of dimensionless reaction
$$\Pr$$: Prandtl number
$$\,Le$$: Lewis number
$$Ec$$: Eckert number
$$P_{e}$$: Peclet number
$L_{b}$: Bioconvection Lewis number
$C_{f}$: Skin friction coefficient
$Nu_{x}$: Local Nusselt number
$Sh_{x}$: Local Sherwood number
$$k$$: Thermal conductivity $$\left( {{\text{W/k}}\;{\text{m}}} \right)$$
$c$: Size of needle

## Greek symbols

$\varphi$: Volume fraction of solid nanoparticles
$$\theta$$: Dimensionless temperature
$$\phi$$: Dimensionless concentration
$\rho_{f}$: Density of fluid $\left( {{\text{kg/m}}^{3} } \right)$
$\rho_{m}$: Density of motile microorganisms $\left( {{\text{kg/m}}^{3} } \right)$
$\varepsilon$: Velocity ratio parameter
$$\mu$$: Dynamic viscosity $$\left( {{\text{kg/m}}^{2} \;{\text{s}}} \right)$$
$\eta$: Variable of transformation
$$\upsilon$$: Kinematic viscosity $$\left( {{\text{m}}^{2} {\text{/s}}} \right)$$

## References

[1] Choi, S. U. S. Enhancing thermal conductivity of fluids with nanoparticles, in Developments and Applications of Non-Newtonian Flows, FED, edited by D. A. Siginer and H. P. Wang (ASME, New York, 1995), vol. 231/MD-vol. 66, 99–105.

[2] Khan MWA, Khan MI, Hayat T, Alsaedi A (2018). Entropy generation minimization (EGM) of nanofluid flow by a thin moving needle with nonlinear thermal radiation. Phys. B.

[3] Salleh SNA, Bachok N, Arifin NM, Ali FM, Pop I (2018). Magnetohydrodynamics flow past a moving vertical thin needle in a nanofluid with stability analysis. Energies.

[4] Waini I, Ishak A, Pop I (2019). Hybrid nanofluid flow and heat transfer past a vertical thin needle with prescribed surface heat flux. Int. J. Numer. Methods Heat Fluid Flow.

[5] Gul T, Khan MA, Noman W, Khan I, Abdullah Alkanhal T, Tlili I (2019). Fractional order forced convection carbon nanotube nanofluid flow passing over a thin needle. Symmetry.

[6] Lee LL (1967). Boundary layer over a thin needle. Phys. Fluids.

[7] Narain JP, Uberoi MS (1972). Combined forced and free-convection heat transfer from vertical thin needles in a uniform stream. Phys. Fluids.

[8] Chen, J. L. S., & Smith, T. N. Forced convection heat transfer from nonisothermal thin needles (1978).

[9] Wang CY (1990). Mixed convection on a vertical needle with heated tip. Phys. Fluids A.

[10] Souayeh B, Reddy MG, Sreenivasulu P, Poornima T, Rahimi-Gorji M, Alarifi IM (2019). Comparative analysis on non-linear radiative heat transfer on MHD Casson nanofluid past a thin needle. J. Mol. Liq..

[11] Ahmad S, Arifin NM, Nazar R, Pop I (2008). Mixed convection boundary layer flow along vertical thin needles: Assisting and opposing flows. Int. Commun. Heat Mass Transf..

[12] Mabood F, Nayak MK, Chamkha AJ (2019). Heat transfer on the cross flow of micropolar fluids over a thin needle moving in a parallel stream influenced by binary chemical reaction and Arrhenius activation energy. Eur. Phys. J. Plus.

[13] Khan I, Khan WA, Qasim M, Afridi I, Alharbi SO (2019). Thermodynamic analysis of entropy generation minimization in thermally dissipating flow over a thin needle moving in a parallel free stream of two Newtonian fluids. Entropy.

[14] Hamid A (2020). Terrific effects of Ohmic-viscous dissipation on Casson nanofluid flow over a vertical thin needle: buoyancy assisting & opposing flow. J. Market. Res..

[15] Tlili, I., Nabwey, H. A., Reddy, M. G., Sandeep, N., & Pasupula, M. Effect of resistive heating on incessantly poignant thin needle in magnetohydrodynamic Sakiadis hybrid nanofluid. *Ain Shams Eng. J*. (2020).

[16] Ramesh GK, Shehzad SA, Izadi M (2020). Thermal transport of hybrid liquid over thin needle with heat sink/source and Darcy–Forchheimer porous medium aspects. Arab. J. Sci. Eng..

[17] Hamid A, Khan M (2020). Thermo-physical characteristics during the flow and heat transfer analysis of GO-nanoparticles adjacent to a continuously moving thin needle. Chin. J. Phys..

[18] Buongiorno J (2006). Convective transport in nanofluids. J. Heat Transf..

[19] Tiwari RK, Das MK (2007). Heat transfer augmentation in a two-sided lid-driven differentially heated square cavity utilizing nanofluids. Int. J. Heat Mass Transf..

[20] Nield DA, Kuznetsov AV (2009). The Cheng-Minkowycz problem for natural convective boundary-layer flow in a porous medium saturated by a nanofluid. Int. J. Heat Mass Transf..

[21] Kuznetsov AV, Nield DA (2013). The Cheng-Minkowycz problem for natural convective boundary layer flow in a porous medium saturated by a nanofluid: A revised model. Int. J. Heat Mass Transf..

[22] Khan A, Shah Z, Alzahrani E, Islam S (2020). Entropy generation and thermal analysis for rotary motion of hydromagnetic Casson nanofluid past a rotating cylinder with Joule heating effect. Int. Commun. Heat Mass Transf..

[23] Islam S, Khan A, Kumam P, Alrabaiah H, Shah Z, Khan W, Zubair M, Jawad M (2020). Radiative mixed convection flow of maxwell nanofluid over a stretching cylinder with joule heating and heat source/sink effects. Sci. Rep..

[24] Tlili I, Khan WA, Ramadan K (2019). MHD flow of nanofluid flow across horizontal circular cylinder: steady forced convection. J. Nanofluids.

[25] Khan WA, Aziz A, Uddin N (2013). Buongiorno model for nanofluid Blasius flow with surface heat and mass fluxes. J. Thermophys. Heat Transf..

[26] Xu H, Fan T, Pop I (2013). Analysis of mixed convection flow of a nanofluid in a vertical channel with the Buongiorno mathematical model. Int. Commun. Heat Mass Transf..

[27] Rahman, M. M., Rosca, A. V., & Pop, I. Boundary layer flow of a nanofluid past a permeable exponentially shrinking surface with convective boundary condition using Buongiorno’s model. *Int. J. Numer. Methods Heat Fluid Flow* (2015).

[28] Ghiasi EK, Saleh R (2019). Analytical and numerical solutions to the 2D Sakiadis flow of Casson fluid with cross diffusion, inclined magnetic force, viscous dissipation and thermal radiation based on Buongiorno’s mathematical model. CFD Lett..

[29] Kuznetsov AV (2010). The onset of nanofluid bioconvection in a suspension containing both nanoparticles and gyrotactic microorganisms. Int. Commun. Heat Mass Transf..

[30] Kuznetsov AV (2011). Nanofluid bioconvection in water-based suspensions containing nanoparticles and oxytactic microorganisms: Oscillatory instability. Nanoscale Res. Lett..

[31] Mallikarjuna B, Rashad AM, Chamkha AJ, Abdou M (2018). Mixed bioconvection flow of a nanofluid containing gyrotactic microorganisms past a vertical slender cylinder. Front. Heat Mass Transf..

[32] Uddin MJ, Alginahi Y, Beg OA, Kabir MN (2016). Numerical solutions for gyrotactic bioconvection in nanofluid-saturated porous media with Stefan blowing and multiple slip effects. Comput. Math. Appl..

[33] Amirsom NA, Uddin MJ, Ismail AIM (2018). MHD boundary layer bionanoconvective non-Newtonian flow past a needle with Stefan blowing. Heat Transf. Asian Res..

[34] Khan WA, Rashad AM, Abdou M, Tlili I (2019). Natural bioconvection flow of a nanofluid containing gyrotactic microorganisms about a truncated cone. Eur. J. Mech. B Fluids..

[35] Zohra, F. T., Uddin, M., Basir, F., & Ismail, A. I. M. Magnetohydrodynamic bio-nano-convective slip flow with Stefan blowing effects over a rotating disc. *Proc. Inst. Mech. Eng. Part N J. Nanomater. Nanoeng. Nanosyst*. (2019)

[36] Alwatban AM, Khan SU, Waqas H, Tlili I (2019). Interaction of Wu’s slip features in bioconvection of Eyring Powell nanoparticles with activation energy. Processes.

[37] Kumar A, Sugunamma V, Sandeep N, Jv RR (2019). Impact of Brownian motion and thermophoresis on bioconvective flow of nanoliquids past a variable thickness surface with slip effects. Multidiscip. Model. Mater. Struct..

[38] Liao SJ (1999). Explicit totally analytic approximate solution for blasius viscous flow problems. Int. J. Non-Linear Mech..

[39] Liao SJ (2010). An optimal homotopyanalysis approach for strongly nonlinear differential equations. Commun. Nonlinear Sci. Numer. Simul..

[40] Amirsom NA, Uddin MJ, Ismail AIM (2019). MHD boundary layer bionanoconvective non-Newtonian flow past a needle with Stefan blowing. Heat Transf.-Asian Res..

[41] Soid SK, Ishak A, Pop I (2017). Boundary layer flow past a continuously moving thin needle in a nanofluid. Appl. Therm. Eng..

[42] Ahmad R, Mustafa M, Hina S (2017). Buongiorno's model for fluid flow around a moving thin needle in a flowing nanofluid: A numerical study. Chin. J. Phys..

[43] Waini I, Ishak A, Pop I (2020). Transpiration effects on hybrid nanofluid flow and heat transfer over a stretching/shrinking sheet with uniform shear flow. Alex. Eng. J..

